# Under-ice environment observations from a remotely operated vehicle during the MOSAiC expedition

**DOI:** 10.1038/s41597-025-05223-1

**Published:** 2025-06-05

**Authors:** Philipp Anhaus, Christian Katlein, Stefanie Arndt, Daniela Krampe, Benjamin A. Lange, Ilkka Matero, Evgenii Salganik, Marcel Nicolaus

**Affiliations:** 1 Alfred-Wegener-Institut Helmholtz-Zentrum für Polar- und Meeresforschung, Bremerhaven, Germany; 2 German Aerospace Center (DLR e. V.), Institute for the Protection of Maritime Infrastructures, Bremerhaven, Germany; 3 University of Hamburg, Institute of Oceanography, Hamburg, Germany; 4 Regionalverband Ruhr, Essen, Germany; 5 Norwegian Polar Institute, Tromsø, Norway; 6 Norwegian Geotechnical Institute, Oslo, Norway; 7 Svalbard Integrated Arctic Earth Observing System Knowledge Centre, Longyearbyen, Svalbard Norway

**Keywords:** Cryospheric science, Acoustics, Marine biology, Physical oceanography, Cryospheric science

## Abstract

Changes in the Arctic sea-ice cover affect the planet’s energy budget, atmospheric and oceanic circulation patterns as well as the ecosystem associated with this unique habitat. Interdisciplinary observations at the interfaces between sea ice and ocean are crucial to better understand the driving processes and bio-physical linkages in this coupled system. During the MOSAiC expedition 2019/2020 to the Arctic Ocean, we used a remotely operated vehicle (ROV) underneath drifting sea ice throughout an entire year. The main objective was to measure physical, chemical, and biological parameters across different surface and sea-ice types while the dive missions were optimized to retrieve optical properties and sea-ice bottom topography. All parameters were measured synchronously, enabling the quantification of their relationships and spatial and temporal variability. In addition, visual documentation of the under-ice environment and the permanently on-ice deployed instrumentation was performed. Overall, we completed more than 80 surveys covering all seasons and various sea-ice and surface conditions. Here, we present all available data, allowing for a year-round comprehensive picture of the under-ice environment.

## Background & Summary

Under-ice surveys using remotely operated vehicles (ROVs) provide a unique access to the under-ice environment not available with traditional methods. Inaccessible, undersampled, and heterogeneous ice-covered areas, such as very thin, newly formed ice, and open and refreezing leads, can be studied on a large scale and in a reasonably short operation time. Furthermore, in-situ measurements of sea ice typically impact the surface conditions and sometimes entail destructive methods like drilling or ice-coring. These impacts make the collection of time series challenging. To minimize the disturbances, it is therefore beneficial to use robotic platforms such as ROVs and autonomous underwater vehicles (AUVs). ROVs and AUVs are platforms that can host an array of interdisciplinary sensors to simultaneously measure physical, biological, and chemical parameters underneath the drifting sea ice enabling non-destructive, efficient, and comprehensive data collection along continuous dive lines. Sophisticated and long-term field observations of those parameters are required to improve our understanding of the Arctic sea-ice cover. Those observations can be used to guide the development of large-scale models and to set model parameters to foster our understanding on how small-scale process models with different levels of complexity and coupling can help to advance predicting Arctic climate change. In this context, the parameterization of the processes at the interfaces between the ocean, sea ice, and atmosphere, such as heat, salt, and gas exchange is of great importance and depends on the availability of field observations.

Within the last decade, ROVs and AUVs have been operated during many expeditions to the Arctic and Antarctic. ROVs were used to investigate the interaction of solar radiation with different sea-ice types and surface conditions such as ice thickness, snow depth, and melt-ponds^1–8^. These radiation measurements and the resulting improved understanding of physical sea-ice properties have also expanded our knowledge of the interaction between surface radiation and sea-ice associated biomass^9–18^. Further biological, biogeochemical, and oceanographic properties were also measured using ROVs^19–23^, AUVs^24–26^, and similar platforms^27^. Sea-ice draft measurements, which can be converted to ice thickness and are therefore a crucial parameter that determines, e.g., the fluxes between the ocean and the atmosphere, were also collected using ROVs^28^, AUVs^29,30^, and submarines^31–33^.

During the above mentioned expeditions, short (hours to few days) or seasonal (1-2 months) ROV and AUV surveys on the same ice floes were performed. The aim of such shorter surveys was to study several sea-ice floes in a larger region and to cover different sea-ice and surface conditions in larger spatial areas. While such efforts enable the description of the seasonal evolution of light transmittance of different sea-ice types, this sampling procedure does not allow for a seasonal or year-round and detailed small-scale analysis of a single sea-ice floe. So far, the seasonal evolution is mostly discussed based on stationary radiation stations, which represent one particular site^34,35^, although the spatial heterogeneity of sea-ice properties is widely described and discussed^2,7^.

**Fig. 1:**
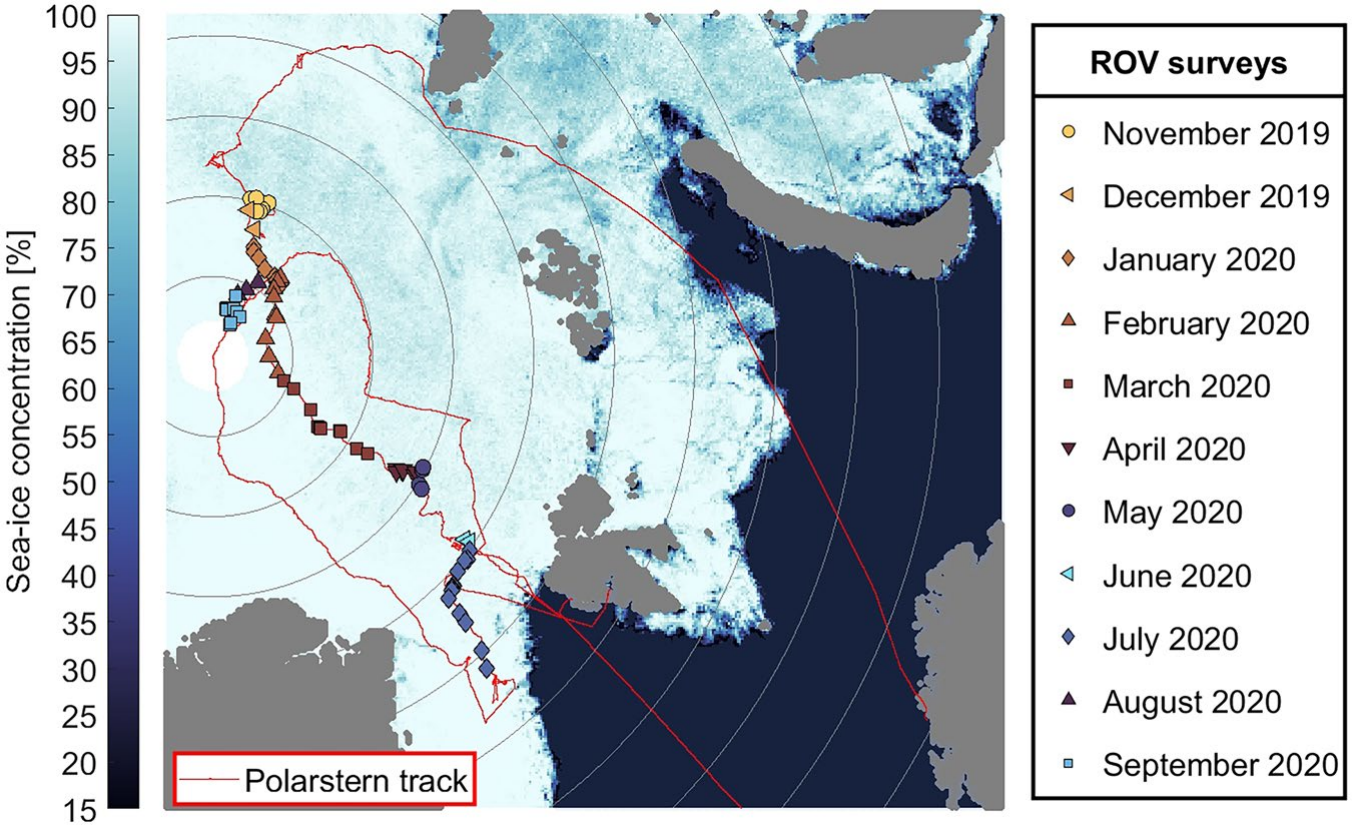
Remotely operated vehicle (ROV) survey locations (colored markers) along the expedition and drift track of the research vessel *Polarstern* during the MOSAiC expedition (red line). The color coding of the ROV locations corresponds to the timing of each respective ROV station. The background is the sea-ice concentration obtained by the Advanced Microwave Scanning Radiometer 2 (AMSR2) on 01/03/2020^95^.

Year-round observations of the properties and processes at the interface of and within the sea ice^36,37^, the ocean^37,38^, and the ecosystem^39^ were conducted during the Multidisciplinary drifting Observatory for the Study of Arctic Climate (MOSAiC) expedition of the research vessel *Polarstern*^40^. The observations were obtained along the Transpolar Drift from north of the Laptev Sea to the Fram Strait from November 2019 to September 2020 (Fig. 1). The ROV-based work during the MOSAiC expedition documented, for the first time, the under-ice environment for all seasons and various sea-ice and surface conditions connecting the sea ice, the ocean, and the ecosystem. The main objective of this work was to collect data that is required to be able to address the scientific gap of transitioning from regional parametrizations to more detailed process-level studies at the scale of individual floes. This includes sea-ice bottom topography, sea-ice optical properties, upper-ocean physical properties, bio-optical and biogeochemical water properties as well as video footage and images. In the following, we describe the ROV operations, present the comprehensive dataset, show exemplary data and results, discuss uncertainties and perform validations.

## Methods

### ROV system and sites

**Fig. 2:**
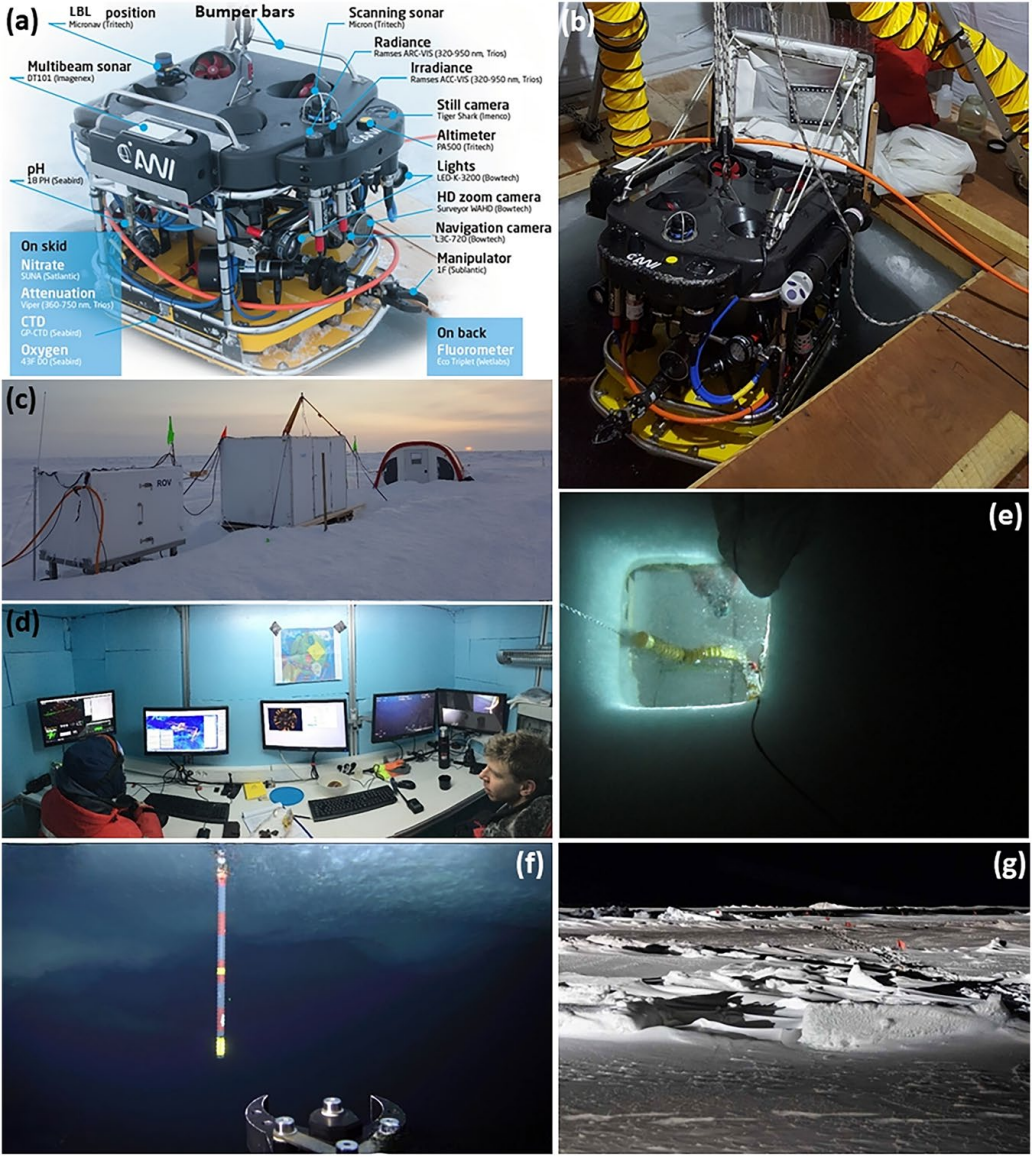
(**a**) Remotely operated vehicle (ROV) with annotated sensors and their positions on the vehicle^28^, (**b**) ROV deployment with the attached zooplankton net through the ROV ice access hole in a tent, (**c**) ROV site 3.0 consisting of the (from left to right) power distribution hub, the ROV control cabin (white hut), and the tent covering the ROV hole as of 14/03/2020, (**d**) inside view of the ROV cabin (photo credit: Steven Fons, NASA), (**e**) upward-looking still image taken below the ROV hole, (**f**) screenshot of the main camera showing a ROV marker pole used for orientation in the water underneath the sea ice, (**g**) photograph of the ROV study area on 01/11/2019 showing the under-ice marker positions indicated by the red flags on the ice.

During the MOSAiC expedition, a M500 ROV^28^ (Ocean Modules, Åtvidaberg, Sweden, Fig. 2a,b) with an interdisciplinary sensor payload was used to collect data describing the under-ice environment. This ROV was specifically designed for operations underneath sea ice in the polar regions. Due to its compact size and limited weight it is portable by two people, can be deployed directly from the ice and can be transported by research helicopters as well as smaller fixed wing aircrafts. This allows the ROV to be operated within a wide range of mission scenarios with varying and reduced logistical support infrastructure, e. g., being independent from a large mothership. Compared to industry ROVs, this ROV can be operated by scientists after a short period of introduction. Based on its eight thrusters and compact size, it is highly maneuverable allowing the operation and taking measurements very close to the ice-ocean interface even under undisturbed sea ice to investigate small scale gradients and ocean stratigraphy. Its attached bumper bars protect the sensors and make collision with the sea ice bearable. Deformed features of the under-ice environment such as pressure ridges can cause the ROV to get stuck. Thus, this ROV is trimmed negatively in such that it sinks when it gets stuck or the system fails. This makes it easier to recover, e.g., it can be dragged back to the surface manually.

In this work, we focus on the description of the specific operations and measurements during the more than 80 scientific surveys that we conducted. The locations of the respective ROV surveys are shown in Fig. 1 and details are provided in Tables 1 and 2. Each survey was tagged using an event label, e.g., PS122/1_5-62, consisting of the expedition number of *Polarstern* “PS122”, the expedition leg number “1”, the week number of the expedition “5”, and the cast number “62”. The MOSAiC (=PS122) expedition was separated into five legs each spanning approximately 2-3 months. Additionally, a survey ID for the individual ROV surveys was used, e.g., when several ROV surveys were conducted during the same day. More detailed information are described in the respective chapters of the expedition reports for the individual expedition legs^41–45^.

**Table 1:** Overview of remotely operated vehicle (ROV) survey missions during the MOSAiC expedition 2019/2020.

Survey date	Survey ID	Event label	Survey start [UTC]	Survey end [UTC]	Survey duration [HH:MM]	Survey distance [m]	Survey missions
02/11/2019	1	PS122/1_5-62	07:20	07:45	00:24	—	Fix camera issues, test position, check data streams, orientation survey
05/11/2019	1	PS122/1_6-16	05:32	08:18	02:45	—	Multibeam, ice type survey
06/11/2019	1	PS122/1_6-31	01:27	02:31	01:04	4 702	Multibeam settings, deploy fishnet rope
10/11/2019	1	PS122/1_6-118	07:27	09:41	02:14	5 531	Net trawls, algae sampling
12/11/2019	1	PS122/1_7-18	06:29	09:31	03:02	6 138	Multibeam
13/11/2019	1	PS122/1_7-55	07:02	09:55	02:52	1 474	Media surveys
19/11/2019	1	PS122/1_8-125	09:07	10:27	01:20	2 361	Engineering tests
26/11/2019	1	PS122/1_9-22	09:35	11:37	02:02	3 507	Multibeam
06/12/2019	1	PS122/1_10-113	17:17	12:42	18:25	2 910	24 h net trawls
30/12/2019	1	PS122/2_18-10	12:33	13:05	00:32	—	Engineering test
31/12/2019	1	PS122/2_18-19	07:58	10:50	02:52	4 100	Multibeam, orientation survey
04/01/2020	1	PS122/2_18-89	07:09	14:00	04:51	13 562	Test sediment trap & deployment, net trawls, deploy fishing line
07/01/2020	1	PS122/2_19-27	06:45	13:14	06:29	12 100	Multibeam, ADCP & CTD Fort ridge documentation
11/01/2020	1	PS122/2_19-115	07:49	12:02	03:13	2 100	Net trawls, deployment sediment traps, explore ridge
14/01/2020	1	PS122/2_20-23	07:01	12:00	04:59	11 750	Recovery sediment trap, multibeam high resolution ridge, reading stakes, ridge ADCP
18/01/2020	1	PS122/2_20-101	07:09	12:45	05:36	5 370	Net trawls, algae sampling, reading stakes, depth profiles
21/01/2020	1	PS122/2_21-36	06:47	13:13	06:26	12 100	Multibeam
25/01/2020	1	PS122/2_21-125	06:41	13:09	06:28	4 200	Net trawls, explore ridge and ADCP, depth profiles
28/01/2020	1	PS122/2_22-45	06:48	10:56	04:08	6 800	Multibeam, inspect turbulence cluster, depth profile, reading stakes, ridge optic
02/02/2020	1	PS122/2_22-107	06:44	11:43	04:59	4 500	Deploy sediment trap ridge, trap mooring deployment in hole, net trawls, grid reconnaissance
04/02/2020	1	PS122/2_23-29	06:47	13:20	06:33	11 400	Multibeam
08/02/2020	1	PS122/2_23-116	07:00	12:16	05:16	3 900	Multibeam, net trawls, ridge optic, depth profile, brinicle inspection, ridge ADCP
11/02/2020	2	PS122/2_24-70	06:50	12:50	06:00	9 700	Multibeam
11/02/2020	4	PS122/2_24-70	06:50	12:50	06:00	9 700	”
15/02/2020	1	PS122/2_24-97	10:36	12:11	01:35	1 200	Net trawls (platelet ice), depth profile, reading stakes
18/02/2020	1	PS122/2_25-44	06:31	12:45	06:14	10 500	Multibeam, document platelet ice
22/02/2020	1	PS122/2_25-104	07:21	11:10	03:49	3 400	Net trawls, depth profiles (upcast upwelling radiation), document platelet ice, deployment sediment traps
25/02/2020	1	PS122/3_29-14	10:40	13:36	02:56	3 481	Multibeam, recovery sediment traps, document platelet ice, reading stakes
29/02/2020	1	PS122/3_29-65	12:13	13:03	00:50	1 010	Multibeam, grid transect, ridge optic, inspect stakes, document platelet ice
07/03/2020	1	PS122/3_30-69	07:19	12:45	05:26	827	Net trawls, reading stakes
10/03/2020	1	PS122/3_31-17	08:07	13:00	04:53	10 485	Multibeam, check radiation station
14/03/2020	1	PS122/3_31-75	08:02	13:53	05:51	8 765	Lead transects: CTD, multibeam, optics, video footage lead
17/03/2020	1	PS122/3_32-11	12:23	14:24	02:01	2 708	Ridge multibeam, FYI optics, radiation station, ridge & lead video footage
18/03/2020	1	PS122/3_32-33	07:52	10:56	03:04	8 675	Multibeam
18/03/2020	1	PS122/3_32-34	12:59	14:32	01:33	3 737	Multibeam
22/03/2020	2	PS122/3_32-78	07:52	13:38	05:46	6 580	Multibeam, net trawls, grid optic, depth profile
24/03/2020	1	PS122/3_33-27	09:28	13:51	04:23	10 841	Multibeam
28/03/2020	1	PS122/3_33-83	08:25	13:38	05:13	7 521	Multibeam, net trawls, depth profile, lead transects
31/03/2020	1	PS122/3_34-20	08:30	13:53	05:23	11 201	Multibeam
07/04/2020	1	PS122/3_35-32	08:16	13:38	05:22	4 325	Net trawls, lead optics, depth profile, searching and checking under-water deployments
11/04/2020	1	PS122/3_35-95	07:57	13:51	05:54	5 503	Net trawls, lead optics, depth profile, searching lost under-water deployments
14/04/2020	1	PS122/3_36-24	07:34	14:06	06:32	13 389	Multibeam
18/04/2020	1	PS122/3_36-112	07:53	09:36	01:43	874	Net trawls
18/04/2020	1	PS122/3_36-125	09:38	13:33	03:55	6 162	Net trawls, lead multibeam & optics, depth profile, lead video footage
21/04/2020	2	PS122/3_37-19	07:35	07:40	00:05	0	—
21/04/2020	1	PS122/3_37-20	08:08	14:05	05:57	11 232	Multibeam
25/04/2020	2	PS122/3_37-108	07:28	13:11	05:43	6 110	Net trawls, lead grid, depth profile, video footage
28/04/2020	1	PS122/3_38-50	08:39	14:17	05:38	12 740	Multibeam, crack optics, video footage
01/05/2020	1	PS122/3_38-85	12:00	14:07	02:07	1 131	Lead transects, recover transponder
02/05/2020	1	PS122/3_38-91	07:36	12:58	05:22	7 015	Net trawls, lead & ridge optics, reading stakes
05/05/2020	1	PS122/3_39-20	07:35	12:58	05:23	14 175	Multibeam
09/05/2020	1	PS122/3_39-77	07:37	14:09	06:32	9 667	Net trawls, lead & ridge optics, depth profile, ridge multibeam
10/05/2020	1	PS122/3_39-111	09:04	09:11	00:07	0	Recover ROV site
10/05/2020	2	PS122/3_39-152	09:11	09:48	00:37	367	Recover ROV site

Abbreviations: ADCP: Acoustic Doppler Current Profiler, GP-CTD: Glider Payload - Conductivity Temperature Depth, FYI: First-Year Ice).

**Table 2:** [*Continuation* Table 1] Overview of remotely operated vehicle (ROV) survey missions during the MOSAiC expedition 2019/2020.

Survey date	Survey ID	Event label	Survey start [UTC]	Survey end [UTC]	Survey duration [HH:MM]	Survey distance [m]	Survey missions
24/06/2020	1	PS122/4_44-162	09:07	11:53	02:46	3 383	Explore surroundings, optics, depth profile, ridge multibeam
24/06/2020	2	PS122/4_44-162	11:59	14:39	02:40	5 467	“
27/06/2020	1	PS122/4_44-191	08:45	10:46	02:01	625	Net trawls, deployment sediment traps, optics, albedo line, depth profile
27/06/2020	2	PS122/4_44-191	11:35	14:17	02:42	2 138	“
27/06/2020	3	PS122/4_44-191	12:10	14:40	02:30	815	“
28/06/2020	1	PS122/4_44-206	11:43	13:22	01:39	2 270	Multibeam, optics
28/06/2020	2	PS122/4_44-206	13:22	14:57	01:35	3 720	“
01/07/2020	1	PS122/4_45-61	08:29	11:13	02:44	6 015	Multibeam, optics, hyperspectral images
01/07/2020	2	PS122/4_45-61	11:14	14:47	03:33	2 511	“
03/07/2020	1	PS122/4_45-129	13:49	15:04	01:15	646	Deployment sediment traps
04/07/2020	1	PS122/4_45-149	08:29	10:06	01:37	728	Net trawls, optics, depth profile, albedo line, recover sediment traps
04/07/2020	2	PS122/4_45-149	10:34	14:16	03:42	4 710	“
07/07/2020	1	PS122/4_46-37	08:49	13:12	04:23	8 447	Multibeam, optics, hyperspectral images, albedo line
07/07/2020	2	PS122/4_46-37	13:12	15:00	01:48	762	“
10/07/2020	1	PS122/4_46-172	08:02	13:58	05:56	2 739	Net trawls, optics, albedo line, hyperspectral images, deployment sediment traps
10/07/2020	2	PS122/4_46-174	17:16	19:46	02:30	1 297	“
10/07/2020	3	PS122/4_46-175	23:24	01:51	02:27	1 374	“
11/07/2020	1	PS122/4_46-176	05:16	07:38	02:22	1 355	Net trawls, optics, depth profile, albedo line
11/07/2020	2	PS122/4_46-177	10:42	12:55	02:13	1 461	Recovery sediment traps
11/07/2020	3	PS122/4_46-177	13:15	14:26	01:11	2 130	Multibeam
14/07/2020	1	PS122/4_47-31	11:59	14:50	02:51	5 890	Multibeam, optics, albedo line, depth profile
19/07/2020	1	PS122/4_47-135	11:29	15:00	03:31	3 060	Net trawls, deployment sediment traps, optics, depth profile
21/07/2020	1	PS122/4_48-4	08:04	14:49	06:45	9 585	Multibeam, optics, albedo line, recovery sediment traps, hyperspectral images
26/07/2020	1	PS122/4_48-213	12:04	15:01	02:57	2 679	Net trawls, optics, albedo line, hyperspectral images
28/07/2020	1	PS122/4_49-105	08:00	12:28	04:28	5 730	Multibeam, optics, albedo line, media footage
25/08/2020	1	PS122/5_59-269	08:40	12:30	03:50	7 568	Multibeam, optics, footage of seal, orientation
29/08/2020	1	PS122/5_59-369	05:30	11:45	06:15	9 161	Multibeam, net trawls, optics, orientation
31/08/2020	1	PS122/5_60-5	09:10	11:40	02:30	2 545	Video footage
01/09/2020	1	PS122/5_60-28	05:00	10:40	05:40	1 339	Multibeam, optics, documentation buoys
01/09/2020	2	PS122/5_60-28	10:40	11:20	00:40	435	Video footage
04/09/2020	1	PS122/5_60-165	04:20	07:30	03:10	4 745	Optics, depth profile
04/09/2020	2	PS122/5_60-166	15:00	16:40	01:40	3 906	Optics
05/09/2020	1	PS122/5_60-167	04:20	12:45	08:25	1 428	Multibeam, net trawls, optics, depth profile, lead
08/09/2020	1	PS122/5_61-35	04:50	10:40	05:50	1 413	Multibeam, optics, depth profile, lead
10/09/2020	1	PS122/5_61-156	04:50	11:00	06:10	1 533	Multibeam, optics, depth profile, lead, ridge
12/09/2020	1	PS122/5_61-200	05:00	11:00	06:00	7 244	Net trawls, optics
15/09/2020	1	PS122/5_62-65	04:45	11:45	07:00	1 477	Multibeam, optics, depth profile, lead, buoys
17/09/2020	1	PS122/5_62-103	05:20	09:20	04:00	9 707	Multibeam, optics, depth profile, lead
17/09/2020	2	PS122/5_62-165	09:20	10:20	01:00	525	Media footage

Abbreviations: ADCP: Acoustic Doppler Current Profiler, GP-CTD: Glider Payload - Conductivity Temperature Depth, FYI: First-Year Ice).

The surveys were performed from different ROV sites which were chosen to be 1) representative, 2) cover the different ice types, 3) be connected to all other measurements, and 4) the likelihood to survive sea-ice dynamic events. At the same time, choosing the sites was limited due to logistical needs such as power, accessibility, and safety. The sites were supplied by power (6 kW) from *Polarstern* by cable to heat the installations and to power the system. Each site consisted of a power distribution hub (left in Fig. 2c), a control cabin (middle in Fig. 2c, interior in Fig. 2d), and a heated tent during the cold periods (right in Fig. 2c). The ROV was lowered into the water through an approximately 1.5 m × 1.5 m wide hole in the ice (Fig. 2b,e) and operated vertically and horizontally in the water column and along the ice bottom with a maximum horizontal range of 300 m and maximum vertical range of 100 m.

**Fig. 3:**
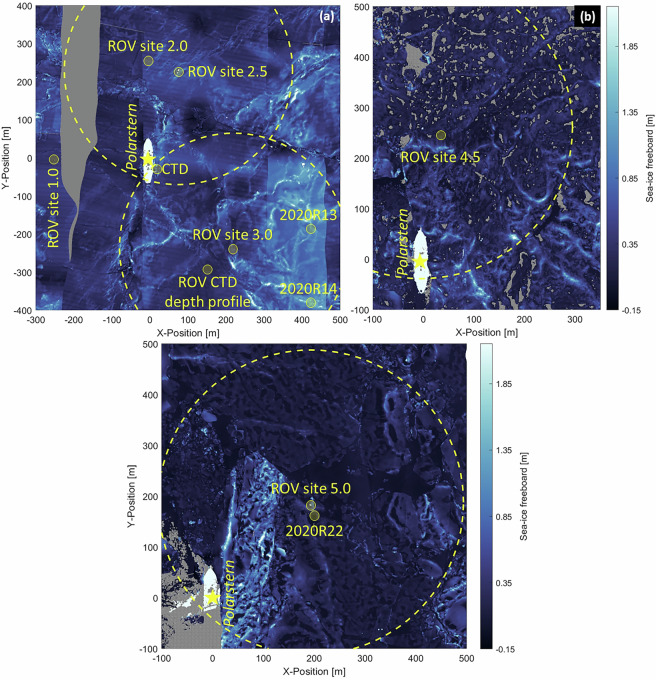
Overview maps of the locations of the ROV sites relative to *Polarstern*. Maps showing the sea-ice freeboard derived from helicopter airborne laser scanning data^64,96,97^. Map on (**a**) 19/11/2019 showing ROV sites 1.0, 2.0, 2.5 and 3.0, (**b**) 22/07/2020 showing ROV site 4.5 surrounded by leads and open melt ponds, (**c**) 15/09/2020 showing ROV site 5.0 surrounded by new and refrozen melt ponds and leads. The yellow circles represent the reach of the ROV, 300 m around the ROV ice access hole. For reference and orientation, other sites are marked where scientific data were acquired (*Polarstern* CTD, radiation stations 2020R13, 2020R14, 2020R22). Grey color in (**a**) indicates that no data is available and in (**b**) and (**c**) refers to open water and melt ponds.

The ROV sites and setups had to be re-located and re-established several times due to sea-ice dynamic events and re-anchoring of *Polarstern*. The ROV site 1.0 was established approximately 250 m off the front of the bow of *Polarstern* next to an active crack (Fig. 3a, 12-19/10/2019). At this site, no scientific data were acquired due to re-location after a sea-ice break-up event during the expedition. The ROV site 2.0 was established in a similar relative location. However, this site was significantly displaced and disconnected from power due to continuing shear in the main shear zone for a extended period of time, until power lines could be re-established and ROV site 2.5 was established (31/10/2019 until 16/12/2019). At this site, a grid was established that spanned a 100 m × 100 m area of undisturbed ice consisting of an array of plastic marker poles. The marker poles (1 m long) were deployed from the surface through drilled holes in the ice and penetrated into the water below (Fig. 2f,g). ROV surveys were performed along these grid lines and in the area between. This grid was chosen to have representative ice conditions of ice that survived the previous summer as mostly level ice and new ice. Surface deformations resulted from the shear events in the first week of the expedition and represented shallow ridge and rafting structures. The grid suffered from several events of small scale deformations, crack openings, and ridge formation. This site had to be abandoned after 16/12/2019, because a crack formed through the ROV tent.

The ROV site 3.0 (Figs. 2c and 3a, 24/12/2019 until 10/05/2020) was selected based on better access to sea-ice ridges and the proximity to other measurements and allowed access to ice similar to the first two deployment sites as well as some parts of the ice and snow thickness transect routes^46^. The ROV hole was placed on a patch of 0.80 m thick ice surrounded by thicker patches. On 28/01/2020, a 100 m × 100 m grid was established on first-year ice to achieve a timeseries of sea-ice optical and draft measurements. The ice thicknesses for this grid ranged from 0.84 m to 2.65 m with a mean of 1.30 m. Within one week after a major ice dynamics event occurred on 11/03/2020, this ice patch was out of reach for the ROV and could no longer be used despite the ROV site remaining stable. During that event, a lead opened at the ROV site and refroze and collected a snow cover over time. On 06/04/2020, a grid on the lead was established. The ice thickness on that day ranged from 0.04 m to 0.61 m with a mean of 0.38 m. Due to logistical reasons, the ROV site except the tent was recovered between 10/05/2020 and 12/05/2020. After a major storm and subsequently severe ice dynamic events, the ROV site 3.0 including the tent was destroyed between 13-14/05/2020.

After a six weeks interruption of supervised scientific efforts on the ice floe, the ROV site 4.0 (20/06/2020 and 23/06/2020) was established. However, at this site, no scientific data were acquired due to re-location after a sea-ice break-up event. After the event, the ROV site 4.5 (24/06/2020 until 28/07/2020) was established (Fig. 3b). The ROV hole was cut through 1.7 m to 1.8 m first-year ice, approximately 20 m away from large ridges and open water. The site had a dry snow cover at the time of deployment but was soon affected and surrounded by melt water from melting snow and forming melt ponds. Melt ponds started to appear on 21/06/2020 and were numerous since 27/06/2020^34^ (grey in Fig. 3b). The ROV control cabin was relocated closer to the ridge and approximately 30 m further away from the ice edge on 24/07/2020.

Since the MOSAiC floe had moved into the Marginal Ice Zone and completely disintegrated by that time, *Polarstern* proceeded northward again to establish a second MOSAiC floe in the vicinity of the North Pole and, thus, could not continue the time series observations of comparable ice in direct vicinity. On this different floe, the ROV site 5.0 (Fig. 3c, 24/08/2020 until 18/09/2020) was established. The ROV hole was cut through 1.4 m thick ice. Ice thicknesses around the area ranged from 0.95 m to 1.40 m. A part of the original measurement field shifted during a shear event between 30/08/2020 and 31/08/2020. The last survey at this site and during the entire MOSAiC expedition was performed on 17/09/2020.

### Operations and datasets

During the MOSAiC expedition, more than 80 surveys were completed focusing on different scientific aspects and data collection. The locations of *Polarstern* at the start time of each ROV survey are illustrated in Fig. 1. The focus of the surveys, highlights encountered during a survey, and actions performed are provided in Tables 1 and 2 and were logged in real-time during each survey by the ROV operators in attention points. The datasets of these logs are referred to in Table 3.

**Table 3:** Published datasets collected using the remotely operated vehicle (ROV) during the MOSAiC expedition 2019/2020^77^.

Parameter	Sensor	Data access
Positioning	LBL	10.1594/PANGAEA.952676
Telemetry	IMU	10.1594/PANGAEA.952676
Single-beam sea-ice draft	Altimeter	10.1594/PANGAEA.951647
Multibeam sea-ice draft	Sonar	10.1594/PANGAEA.971872
Spectral radiation	RAMSES	10.1594/PANGAEA.979856
Spectral attenuation	VIPER	10.1594/PANGAEA.952801
Currents	ADCP	10.1594/PANGAEA.953510
Hydrography	GP-CTD	10.1594/PANGAEA.953910
Fluoresence	ECO triplet	10.1594/PANGAEA.952014
pH	pH sensor	10.1594/PANGAEA.951794
Nitrate	SUNA	10.1594/PANGAEA.953490
Videos	Video camera	10.1594/PANGAEA.953144
Upward-looking still images	Still camera	10.1594/PANGAEA.958183
Attention points		10.1594/PANGAEA.951904
Quicklook plots		10.1594/PANGAEA.952951
Data collection in Ocean Data View (ODV)		mvre.webodv.cloud.awi.de

Technical details and specifications for each parameter and sensor are available^28^. Abbreviations: LBL: Long Base Line, IMU: Inertial Measurement Unit, ADCP: Acoustic Doppler Current Profiler, GP-CTD: Glider Payload - Conductivity Temperature Depth.

The plastic marker poles that were put through drilled holes in the ice (Fig. 2f) were used to co-locate data collected with the ROV with surface data, to ease under-ice navigation of the ROV, and to assess the quality of the ROV position. The markers were installed every 10 m and their positions were measured either using a terrestrial laser scanner (TLS, VZ-1000, RIEGL)^47,48^, by tape measure or by handheld GPSs. Markers had red flags at the surface (Fig. 2g).

**Fig. 4:**
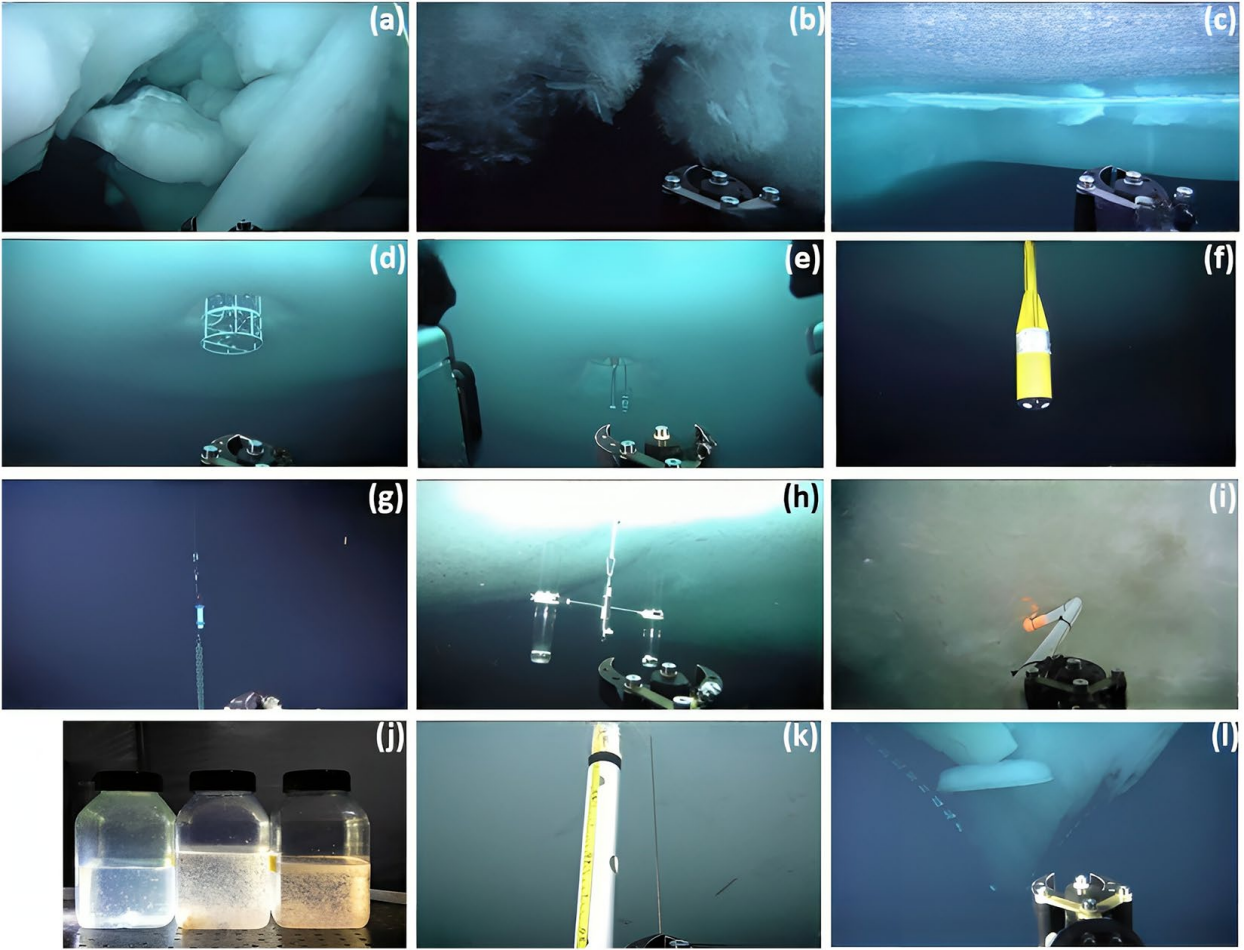
(**a**) Filming ridged sea ice and observe ice-block sizes, (**b**) documenting platelet ice and (**c**) new ice formation, observing (**d**) a CTD cast and (**e**) a Micro Structure Sonde cast at Ocean City, documenting a permanent (**f**) Acoustic Doppler Current Profiler and (**g**) CTD installation, (**h**) inspecting a sediment trap, (**i**) sampling of algae by a suction pump, (**j**) photographs of zooplankton catches from three different depths on 08/02/2020, (**k**) video screenshot of reading sea-ice thickness from stakes, and (**l**) inspecting the hull of *Polarstern*.

The following provides a description of the ROV survey missions. The surveys were mostly performed on two dedicated days during a week. Usually, one complete day was dedicated to multibeam surveys of a large part of the floe, while a second day was spent for ROV nets and other biology related work. Once sunlight returned by mid March, the second day was also used in addition for detailed optical surveys. The intensive program allowed to investigate the spatial variability and the seasonal cycle of sea-ice optical properties, sea-ice bottom topography, bio-physical properties of the uppermost ocean (e.g. 50-100 m), organisms living under the sea ice and in ridge voids as well as their variability across and under various surface features and ice types^36^. This includes in particular:

- High resolution (point spacing of 0.25 m) studies of sea-ice bottom topography and roughness were achieved by extensive (up to 200 000 m^2^) acoustic multibeam sonar surveys^49^. Beyond sea-ice mass balance studies, these data may also be used for observation-derived heat and salinity fluxes at the ice-ocean interface^50^.
- Survey grids (250 m × 250 m) and depth profiles (down to 100 m) to measure solar irradiance and radiance under sea ice. These allow three-dimensional mapping of solar energy fluxes under sea ice. Additional, roll experiments (rolled ROV side-wards from 0^∘^ to 180^∘^ by increments of 20^∘^) allowed to document the geometry of the under-ice light field, including upwelling radiation from the water below. Specific measurements at marker positions were used to co-locate with surface optical measurements, e.g., in-situ surface albedo^51–53^, surface albedo derived from RGB aerial images^54^, and by autonomous radiation stations^34,55^ which provide incoming, reflected, and transmitted radiation.
- Hyperspectral images were recorded to characterize the spatio-temporal variability of bio-physical sea-ice properties^56^.
- Vertical and lateral variability of physical (temperature, salinity), chemical (pH, nitrate, oxygen), and bio-optical (fluorometry, ultra-violet and visible absorbance spectroscopy) water properties were mapped during all surveys.
- Visual documentation of sea-ice conditions from underneath from photography and video surveys allowed to describe and partly quantify the conditions of sea-ice ridges (Fig. 4a), the formation of platelet ice (Fig. 4b^57^), or new ice formation (Fig. 4c^58^).
- Performing under-ice deployments, manipulation, and maintenance of installations, e.g., autonomous (radiation) stations, ice mass balance buoys, sediment traps for faeces collection, inspection and recovery of instrumentation (Fig. 4d–h).
- Sampling of in-ice bottom algae using a suction pump (Fig. 4i) and zooplankton and gypsum vertical distribution in the direct vicinity of the sea ice as analyzed from the ROV towed net during dedicated surveys (Figs. 2b and 4j^39,59^).
- Reading stakes for ice mass balance studies (Fig. 4k^60,61^).

### Sensor data

In this section, we briefly describe which parameters were measured by which sensor.

#### Positioning and telemetry

Deriving a high-quality horizontal position of the ROV under sea ice is a major challenge and also a main source for uncertainty for all data processing and co-location. The X and Y positions of the ROV were measured using an acoustic Long Base Line positioning system (Pinpoint 1500, LinkQuest, San Diego, CA, USA) with an operating frequency of 26.77 kHz to 44.62 kHz. It consists of a transceiver onboard the ROV and three transponders at a time which were deployed on 5 m long chains through the sea ice at different horizontal distances from the ROV hole. One transponder was always deployed in the hole itself. The positions of the transponders were measured, similarly as the marker poles, and converted to the ROV coordinate system. Due to the high latitude and glitches in the survey software’s coordinate conversions, the surveys were moved to virtual geographic positions centered around 1° N / 1° E. The survey track was smoothed using a Kalman filter in the Spot.On survey software (Ocean Modules, Åtvidaberg, Sweden) from initial acoustic fixes and cleaned for most obvious outliers. This set-up resulted in a floe-fixed, relative coordinate system (X, Y) with the origin (X=0 m, Y=0 m) at the ROV hole.

During data processing, a quality flag for the position information was introduced based on the time to the closest acoustic fix with “1” indicating good position (fix reached  < = 3 s ago), “2” medium position (fix reached > 3 s and  < = 5 s), and “3” bad position (fix reached  > 5 s). Depending on the scientific aim, a position with quality flag “3” can still be useful. ROV depth was measured by an integrated pressure sensor (Keller A-21Y, Keller AG, Jestetten, Germany) included in the main electronics housing of the ROV. The depth was calibrated during pre-survey procedures, in such that the top side of the ROV (bumper bars, Fig. 2a) was horizontally aligned with the water surface and calibrated to show 0 m. The accuracy of the depth sensor is 0.10 m. A second depth measurement was derived from the attached GP-CTD sensor with a higher accuracy. ROV telemetry (roll, pitch, heading) was measured with an onboard inertial measuring unit (Microstrain, Williston, VT, USA) with three axis accelerometer, magnetometer, and gyroscope. Due to the vicinity to the magnetic pole, the heading information was useless most of the time. Hence, the heading information was derived as course over ground as retrieved from the filtered acoustic tracking in post processing. The maximum speed of the ROV is approximately 4 m s^−1^. However, most optics surveys were usually performed at a constant speed of approximately 0.25 m s^−1^.

#### Sea-ice draft

The sea-ice thickness in the Arctic Ocean ranges between a few centimeters for newly formed ice, several meters for highly deformed ice and up to 47 m for pressure ridges^33,62^. Our objective was to measure sea-ice draft that can be used as a proxy for sea-ice thickness using a conversion factor that depends on the local hydrostatic equilibrium based on the snow mass and the isostasy principles. Due to the relatively small spatial scales of the ROV surveys, the multibeam can provide ice draft measurements on the scales where the balance of gravity and buoyancy forces acting on snow and ice is satisfied, whereas for tens to hundreds of meters it is not^63^. Further advantages in using ROV surveys are smaller uncertainties compared to remote sensing methods and more details and coverage as well as less time consumed compared to collecting surface measurements.

Here, we used two acoustic sonars attached to the ROV that measure the distances between their sensor heads and the sea-ice underside. Subtracting those distances from the ROV depth in the water yields the sea-ice draft. By performing extensive ROV surveys several hours long, we retrieved very detailed maps of spatial and temporal variability in sea-ice draft, although for a relatively small area.

A single-beam upward-looking sonar altimeter (PA500, Tritech, Aberdeenshire, UK) with an operating frequency of 500 kHz was used to collect sea-ice draft point measurements. The derived draft data was not corrected for pitch and roll of the ROV. An offset between the ROV bumper bars and the altimeter of 0.11 m is accounted for in the draft data. An uncertainty of 0.10 m in the draft data can occur due to the accuracy of the ROV depth sensor. The range for the distance from the single-beam sonar to the sea-ice underside is 0.10-10.0 m.

The three-dimensional geometry of the sea-ice underside has been mapped in a lawnmower pattern using a high-resolution multibeam sonar (DT101, Imagenex Technology Corp., Port Coquitlam, BC, Canada) attached to the ROV (Fig. 2a). The area covered resulted mostly from the dive time available on the respective day. Standard multibeam surveys were performed in a dive depth of 20 m while high-resolution surveys in a depth of 10 m. The maximum range for the distance between the multibeam sonar and the sea-ice underside is 70 m. Horizontal distances between individual dive lines were between 20 m and 25 m to achieve some 30% overlap of the area scanned by the outer beams of neighboring lines. The system consists of a multibeam sonar, an integrated sound velocity probe, and a motion reference unit. The operating frequency was 240 kHz. The sonar was used with 480 beams emitted at the same time with a swath width (nominal beam geometry) of 120° (across track) × 3° (along track) and an effective beam width of 0.75°. The angular resolution resulting from the chosen number of beams and the sector size was 0.25°. The ping rate was automatically set and was based on the range setting, numbers of beams selected and specification of the computer system used for operation. Those settings resulted in a mean ping rate of 9.37 Hz for all surveys. A constant sound speed of 1436 m s^−1^ was applied to allow consistent processing of the data within the highly stratified mixed layer of the Arctic Ocean where we assumed no significant distortions due to density. Returns from three pings were averaged to derive the distance from the multibeam to the ice underside. Subtracting the distance to the ice from the ROV depth yielded the sea-ice draft. Data were processed using the CARIS HIPS and SIPS software (Teledyne CARIS v10) applying swath and subset editors for data cleaning. The horizontal spacing between measurements for standard surveys is 0.5 m and for high-resolution surveys 0.1 m. The vertical offset of 0.05 m between the ROV depth calibration reference (bumper bars, Fig. 2a) and the multibeam is accounted for in the draft data. Absolute calibration of the depth used for the multibeam data processing was achieved by cross-correlating the GP-CTD pressure sensor to the ROV internal pressure sensor.

While the single-beam sonar measurements provide detailed high resolution sea-ice draft along the ROV dive track, the multibeam sonar maps the three-dimensional geometry of the sea-ice underside. Combining multibeam measurements with airborne^64^ or terrestrial surface laser scanning^47^ results in full three-dimensional sea-ice thickness fields^65^. The single-beam sonar data is streamed live to the operator and, thus, is helpful for navigation and avoiding collision with sea ice and obstacles.

#### Optical properties

Spectral planar irradiance and radiance transmitted through the sea ice into the ocean below were measured by a RAMSES-ACC (Advanced Cosine Collector) and a RAMSES-ARC (Advanced Radiance Collector) hyper-spectral radiometer (TriOS Mess- und Datentechnik GmbH, Rastede, Germany), respectively. The radiometers were mounted upward looking on the ROV (Fig. 2a). The 180° opening angle of the irradiance sensor results in a large footprint radius on the surface. Thus, those measurements are used for energy budget calculations and to study biological processes depending on the under-ice light conditions. The radiance sensor has a much narrower opening angle of 7°. Thus, the effects of small-scale variations at the surface such as varying snow depth on the sea-ice optical properties can be detected. In addition, we measured incident planar irradiance on the surface using a tripod on top of the control cabin (Fig. 2c). Those measurements were used to calculate transmittance and transflectance through normalization of transmitted irradiance and radiance by the incident irradiance, respectively^7^. The spectral resolution of each radiometer is 3.3 nm, which was interpolated to a common wavelength grid with 1 nm spacing^35^. All parameters are also provided as integrated fluxes over the photosynthetically active radiation (PAR, 400-700 nm) range and over the entire wavelength range (broadband, total, 320-950 nm). Incident and transmitted irradiance as well as transmitted radiance are also provided as absolute photon fluxes integrated over the PAR range. The spectral measurements were multiplied with the respective PAR wavelengths *λ* and with a constant including the Avogadro number N_*A*_ = 6.022 × 10^23^ *mol*^−1^, the Planck constant *h* = 6.63 × 10^−34^ *J* *s*, and the speed of light *c* = 2.998 × 10^8^ *m* *s*^−1^ as follows (e. g., for irradiance): $absolute\,photon\,flux,irradiance=\frac{spectral\,irradiance\times \lambda \times 1\times {10}^{-9}}{h\times c\times {N}_{A}\times 1\times {10}^{-6}}$.

The magnitude and spectral shape of the transmittance strongly depend on the sea-ice properties. Attenuation of the magnitude is dominated by scattering between the pure ice and the inclusions within the ice, such as brine, air bubbles, or solid salts^66–68^, the surface conditions such as snow cover^1,2,69^, a surface scattering layer^70^ or melt ponds^2,8^, and on inclusions within the ice^66^. The spectral shape is dominated by absorption due to the ice and chlorophyll associated with biology^71^.

Absorbance and spectral absorption coefficient parameters of light in the water column were measured by a spectral transmissometer (VIPER G2, TriOS Mess-und Datentechnik GmbH, Rastede, Germany) mounted in the sensor skid of the ROV (Fig. 2a). The path length was 0.25 m and wavelengths were selected between 360-750 nm. The calibration was performed by the manufacturer. Spectral absorption coefficients are used to describe the true coloration of water which is caused by dissolved substances.

An underwater hyperspectral imager (UHI, Ecotone AS, Trondheim, Model 4) camera with a radiometric calibration by the manufacturer was attached to the ROV (not installed on the photo in Fig. 2a,b). Hyperspectral surveys were conducted on 01/07/2020, 07/07/2020, 10/07/2020, 21/07/2020 and 26/07/2020 covering level first-year ice and an adjacent ridge flank. The UHI was mounted so that the top of the imager was aligned with the surface of the ROV, and to ensure proper along-track and across-track scanning orientation with the direction of travel. The UHI has a field-of-view of 60° across-track and approximately 0.4° along -track, with a spectral range between 381 nm and 749 nm, with 214 spectral bands and a spectral resolution of approximately 1.7 nm. The UHI integration allowed live data and video feeds to the ROV operation computer during surveys. Spectral quality and features could be assessed and noted in real time with a live stream of data and video. UHI surveys were designed to characterize the biophysical habitat properties of level first-year ice, within the main optical ROV survey area, and the flank of an adjacent ridge which was physically sampled on a weekly basis during July^56,72^.

#### Physical oceanographic properties

Conductivity, temperature, and pressure were measured by a Glider Payload CTD (SBE GP-CTD, Sea-Bird Scientific, Bellevue, WA, USA). Oxygen frequency was measured by an oxygen optode (SBE 43F DO, Sea-Bird Scientific). Both instruments were mounted in the sensor skid of the ROV (Fig. 2a). The Gibbs SeaWater (GSW) Oceanographic Toolbox of TEOS-10^73^ in MATLAB was used to derive depth, salinity, absolute salinity, in-situ and potential densities, and sound speed from the GP-CTD as displayed in Table 4. Oxygen frequency was converted into dissolved oxygen concentration using the OOI Level 2 Fast Dissolved Oxygen core data product^74^. Geographic positions, latitude and longitude, used for the derivatives and conversions were measured by GPS on *Polarstern* at the start time of the respective ROV surveys. In November and beginning of December, instead of the GP-CTD, a RBR-CTD was used. This data is not available in the presented dataset.

**Table 4:** Conversion of parameters measured with the Glider Payload CTD and oxygen optode to other hydrographic parameters and dissolved oxygen.

Parameter	Symbol	MATLAB routine	Unit
Salinity	S	gsw_SP_from_C (10 * C, T, P)	—
Absolute salinity	S_abs	gsw_SA_from_SP (S, P, lon, lat)	g kg^−1^
Conservative temperature	T_c	gsw_CT_from_t (S_abs, T, P)	°C
Density	*ρ*	1 / gsw_specvol (S_abs, T_c, P)	kg m^−3^
Potential density	*ρ*_*pot*	gsw_pot_rho_t_exact (S_abs, T, P, 0)	kg m^−3^
Sound speed	c_sound	gsw_sound_speed (S_abs, T_c, P)	m s^−1^
Oxygen	O	OOI L2 data product DOCONCF^74^	*μ* mol l^−1^
Depth	d	gsw_z_from_p (P, lat)	m

Details can be found in the MATLAB processing scripts^75^. Abbreviations: GP-CTD: Glider Payload - Conductivity Temperature Depth, P: Pressure, lon: longitude, lat: latitude.

Measurements of current speed relative to the ROV were performed by a 2 MHz acoustic doppler current profiler (ADCP, Nortek Aquadopp Profiler, Nortek AS, Rud, Norway). The three transducers of the ADCP obtain narrow acoustic beams (here 1.7° ) which is essential for obtaining high resolution data. The ADCP senses the full three-dimensional speed with three beams, all pointing in different directions (Nortek AS, Aquadopp Current Profiler). The speed was measured within 20 cells each the size of 0.5 m. The blanking distance was 0.21 m. A XYZ coordinate system was used, an orthogonal coordinate system relative to the ADCP (instrument reference frame) determined by the head configuration. The head orientation was changed between different surveys (side looking, ice looking) which can be detected from the sensor’s roll measurements.

#### Biological oceanographic properties

A pH sensor (SBE 18, Sea-Bird Scientific, Bellevue, WA, USA) was used to derive pH values in the water column from the sensor voltages by applying a calibration equation given by the manufacturer (Table 5). The calibration coefficients, pHslope (4.597) and pHoffset (2.573 V), were obtained using three calibration pH buffer solutions with known pH values of 4.00, 7.06, and 10.18^75^. Within those solutions, the voltage output of the pH sensor was measured at a temperature of 296.45 K.

**Table 5:** Calibration of parameters measured with the ECO-triplet fluorometer.

Parameter	Equation	Unit
pH	7.0 + Vout - pHoffset / pHslope * GP-CTD temperature (K) * 1.98416E-4	—
Backscatter strength	1.79e-06 (m^−1^ sr^−1^) / cts * (backscatter counts - 48 cts)	m^−1^ sr^−1^
FDOM concentration	0.0906 ppb / ct * (FDOM counts - 46 cts)	ppb
Chlorophyll-a concentration	0.0073 (*μ*g *l*^−1^) / ct * (chlorophyll counts - 49 cts)	*μ*g l^−1^

Details can be found in the MATLAB processing scripts^75^. Abbreviations: cts: counts, GP-CTD: Glider Payload - Conductivity Temperature Depth.

A UV-spectrometer (SUNA V2, Satlantic, Halifax, NS, Canada) mounted in the sensor skid of the ROV measured the concentration of dissolved nitrate and UV-absorbance spectra. The SUNA has a deuterium UV light source and measured along a path with 0.01 m length and in the wavelength range of 190-370 nm. By illuminating the water, the emitted light is absorbed and scattered by particles and molecules along the pre-defined path through the water. The remaining spectrally-resolved light is measured by a photo-spectrometer. The difference between this measurement and a prior baseline reference measurement of pure water results in an absorption spectrum. The relationship between the total measured absorbance and the concentrations of individual components such as nitrate is based on the Beer-Lambert law for multiple absorbers. Using this relationship, the SUNA obtains a best estimate for the nitrate concentration using multi-variable linear regression^76^.

Fluorometric data on chlorophyll-a concentration at 695 nm, dissolved organic matter (FDOM or fCDOM) concentration at 460 nm, and optical backscatter strength at 700 nm were obtained using an ECO-triplet fluorometer (ECO-Puck BBFL2SSC, Wetlabs, Corvallis, OR, USA). The raw signals at the specific wavelengths were measured in counts and converted into the physical parameters using calibration coefficients from the manufacturer and conversion equations displayed in Table 5. The backscatter dark counts are 48, FDOM dark counts 46, and chlorophyll dark counts 49.

The accuracy of water column sensors, such as nitrate, pH or chlorophyll fluorescence heavily depends on post-mission calibration using water samples analyzed in the laboratory. This affects all bio-optical and chemical sensors that are prone to sensor drift and change their response depending on environmental conditions. Those calibrations have not been performed but can be conducted with samples and data that are available within the MOSAiC consortium.

#### Visual documentation

Visual documentation of the under-ice environment includes upward-looking still images taken every 5 s by a photo camera (Tiger Shark, resolution 14 megapixel, Imenco AS, Aksdal, Norway) with internal flash as well as videos recorded by a HD-zoom camera (Bowtech Surveyor WAHD, resolution 2 megapixel, Teledyne Bowtech, Aberdeen, UK) with a 10:1 optical zoom (Fig. 2a,b). An example of an upward-looking image is shown in Fig. 2e. Examples of video screenshots are presented in Figs. 2f and 4a-i,k,l. The time and position of highlights captured in the video footage were commented and logged referred to as attention points (Table 3).

## Data Records

Data and meta data for all ROV surveys are published on PANGAEA with open access^77^. The digital object identifiers (doi) to the respective data are provided in Table 3. The data is freely available in accordance with the MOSAiC data policy. Most data were pre-processed using the MATLAB software^75^ and is provided in ASCII tab-delimited text files (.tab) while videos, video screenshots and quicklook plots, and upward-looking still images are provided in Moving Picture Experts Group (.mpg), Portable Network Graphics (.png), and Joint Photographic Experts Group (.jpg) formats, respectively^77^. In the following, each data file is briefly described including file names, file types, definitions of column headings, and units.

### Positioning and telemetry

The parameters within each file of the **positioning and telemetry** of the ROV are date/time [UTC]; survey ID; distance, relative, X (Dist rel X) [m]; distance, relative, Y (Dist rel Y) [m]; quality flag, position (QF pos); time in seconds (Time) [s] to the closest acoustic fix; depth, water [m]; heading [^°^]; roll [^°^]; and pitch [^°^].

### Sea-ice draft

The parameters within each file of the **single-beam sea-ice draft** are date/time [UTC]; survey ID; distance, relative, X (Dist rel X) [m]; distance, relative, Y (Dist rel Y) [m]; quality flag, position (QF pos); depth, water [m]; distance to sea-ice bottom [m]; and sea-ice draft [m].

The parameters within each data file of the **multi-beam sea-ice draft** are distance, relative, X (Dist rel X) [m]; distance, relative, Y (Dist rel Y) [m]; and sea-ice draft [m]. Additionally, each data file is characterized by event; date/time (start) [UTC]; date/time (end) [UTC]; latitude (start) [^°^]; longitude (start) [^°^]; latitude (end) [^°^]; and longitude (end) [^°^].

### Optical properties

The parameters within each file of the **incident spectral solar irradiance measured above sea ice** are date/time [UTC]; survey ID; distance, relative, X (Dist rel X) [m]; distance, relative, Y (Dist rel Y) [m]; altitude [m] of the sensor above ground; irradiance, incident (Ei) [W m^−2^]; irradiance, incident, photosynthetically active (Ei_PAR) [W m^−2^]; irradiance, incident, photosynthetically active, absolute (Ei_PAR_abs) [*μ*mol m^−2^ s^−1^]; and spectral irradiance, incident at wavelengths 320 nm to 950 nm [W m^−2^ nm^−1^].

The parameters within each file of the **downward spectral solar irradiance as measured in different depths under sea ice (transmitted irradiance)** are date/time [UTC]; survey ID; distance, relative, X (Dist rel X) [m]; distance, relative, Y (Dist rel Y) [m]; quality flag, position (QF pos); depth, water [m]; irradiance, downward (Ed) [W m^−2^]; irradiance, downward, photosynthetically active (Ed_PAR) [W m^−2^]; irradiance, downward, photosynthetically active, absolute (Ed_PAR_abs) [*μ*mol m^−2^ s^−1^]; and spectral irradiance, downward at wavelengths 320 nm to 950 nm [W m^−2^ nm^−1^].

The parameters within each file of the **downward spectral solar radiance as measured in different depths under sea ice (transmitted radiance)** are date/time [UTC]; survey ID; distance, relative, X (Dist rel X) [m]; distance, relative, Y (Dist rel Y) [m]; quality flag, position (QF pos); depth, water [m]; radiance, downward (Ld) [W m^−2^]; radiance, downward, photosynthetically active (Ld_PAR) [W m^−2^]; radiance, downward, photosynthetically active, absolute (Ld_PAR_abs) [*μ*mol m^−2^ s^−1^]; and spectral radiance, downward at wavelengths 320 nm to 950 nm [W m^−2^ nm^−1^ sr^−1^].

The parameters within each file of the **fraction of spectral solar irradiance transmitted through snow and sea ice as measured in different depths under sea ice (transmittance)** are date/time [UTC]; survey ID; distance, relative, X (Dist rel X) [m]; distance, relative, Y (Dist rel Y) [m]; quality flag, position (QF pos); depth, water [m]; transmittance (Tm) [%]; transmittance, photosynthetically active (Tm_PAR) [%]; and spectral transmittance at wavelengths 320 nm to 950 nm [%].

The parameters within each file of the **fraction of spectral solar radiance transmitted through snow and sea ice as measured in different depths under sea ice (transflectance)** are date/time [UTC]; survey ID; distance, relative, X (Dist rel X) [m]; distance, relative, Y (Dist rel Y) [m]; quality flag, position (QF pos); depth, water [m]; transflectance (Tf) [%]; transflectance, photosynthetically active (Tf_PAR) [%]; and spectral transflectance at wavelengths 320 nm to 950 nm [%].

The parameters within each file of the **absorbance and spectral absorption coefficient** of the water column are date/time [UTC]; survey ID; distance, relative, X (Dist rel X) [m]; distance, relative, Y (Dist rel Y) [m]; quality flag, position (QF pos); depth, water [m]; absorbance [AU] at wavelengths 410 nm, 436 nm, 525 nm, 620 nm, and 720 nm; absorption coefficient [m^−1^] at wavelengths 436 nm, 525 nm, and 620 nm; and color, true [mg l^−1^ Pt] at wavelength 410 nm.

### Physical oceanographic properties

The parameters within each file of the **hydrographic data** of the water column are date/time [UTC]; survey ID; distance, relative, X (Dist rel X) [m]; distance, relative, Y (Dist rel Y) [m]; quality flag, position (QF pos); depth, water [m]; pressure, water [dbar]; temperature, water [^∘^C]; conductivity C [mS cm^−1^]; salinity; salinity, absolute [g kg^−1^]; density [kg m^−3^]; oxygen [*μ*mol l^−1^]; oxygen frequency [Hz]; and sound velocity in water [m s^−1^].

The parameters within each file of the **water/ice velocity** are date/time [UTC]; survey ID; distance, relative, X (Dist rel X) [m]; distance, relative, Y (Dist rel Y) [m]; quality flag, position (QF pos); depth, water [m]; cell number; current velocity, relative, X [cm s^−1^]; current velocity, relative, Y [cm s^−1^]; current velocity, relative, Z [cm s^−1^]; current speed [cm s^−1^]; current direction [^°^]; and amplitudes 1, 2, and 3 [cts].

### Biological oceanographic properties

The parameters within each file of the **pH data** in the water are date/time [UTC]; survey ID; distance, relative, X (Dist rel X) [m]; distance, relative, Y (Dist rel Y) [m]; quality flag, position (QF pos); depth, water [m]; pH value; and voltage output (V out) of the pH sensor [mV].

The parameters within each file of the **nitrate and UV-absorbance spectra** in the water are date/time [UTC]; survey ID; distance, relative, X (Dist rel X) [m]; distance, relative, Y (Dist rel Y) [m]; quality flag, position (QF pos); depth, water [m]; nitrate [*μ*mol l^−1^]; nitrogen in nitrate [mg l^−1^]; absorbance at wavelengths 254 nm and 350 nm [AU]; bromide [mg l^−1^]; parameters used to retrieve fitted data including dark value used for fit [cts]; fit aux 1 and 2; fit base 1 and 2; fit RMSE; and integration time factor; temperature, technical, internal (T tech) [^°^C]; voltage, internal (V int) [V]; temperature, technical, spectrometer [^°^C]; temperature, technical, lamp [^°^C]; time in seconds, lamp [s]; voltage, lamp (V lamp) [V]; current, main (I) [mA]; voltage, main (V main) [V]; humidity, relative, technical (RH tech) [%]; and UV-absorbance at wavelengths 189 nm to 396 nm [cts].

The parameters within each file of the **chlorophyll, FDOM, and backscatter** in the water are date/time [UTC]; survey ID; distance, relative, X (Dist rel X) [m]; distance, relative, Y (Dist rel Y) [m]; quality flag, position (QF pos); depth, water [m]; optical backscattering coefficient at 700 nm [m^−1^ sr^−1^]; raw optical backscattering coefficient [cts]; chlorophyll-a at 695 nm [*μ*g / l]; raw chlorophyll-a [cts]; dissolved organic matter [ppb]; and raw dissolved organic matter [cts].

### Visual documentation, quicklook plots, and attention points

The parameters within each file of the **videos** are date/time [UTC] at video start; survey ID; video; distance, relative, X (Dist rel X) [m] at video start; distance, relative, Y (Dist rel Y) [m] at video start; quality flag, position (QF pos) at video start; and depth, water [m] at video start.

The parameters within each file of the **video screenshots** are date/time [UTC]; survey ID; and screenshot.

The parameters within each file of the **upward-looking still images** are date/time [UTC]; survey ID; image; distance, relative, X (Dist rel X) [m]; distance, relative, Y (Dist rel Y) [m]; quality flag, position (QF pos); depth, water [m]; pitch [^°^]; and roll [^°^].

The parameters within each file of the **quicklook plots** are date/time [UTC]; survey ID; and quicklook plot.

The parameters within each file of the **logged attention points** are date/time [UTC]; survey ID; distance, relative, X (Dist rel X) [m]; distance, relative, Y (Dist rel Y) [m]; quality flag, position (QF pos); depth, water [m]; identification; and comment.

## Usage Notes

In this section, we show examples of how to use, visualize, and analyze the data. We also present examples of how to approach interpretation of the data.

### Sea-ice draft

**Fig. 5:**
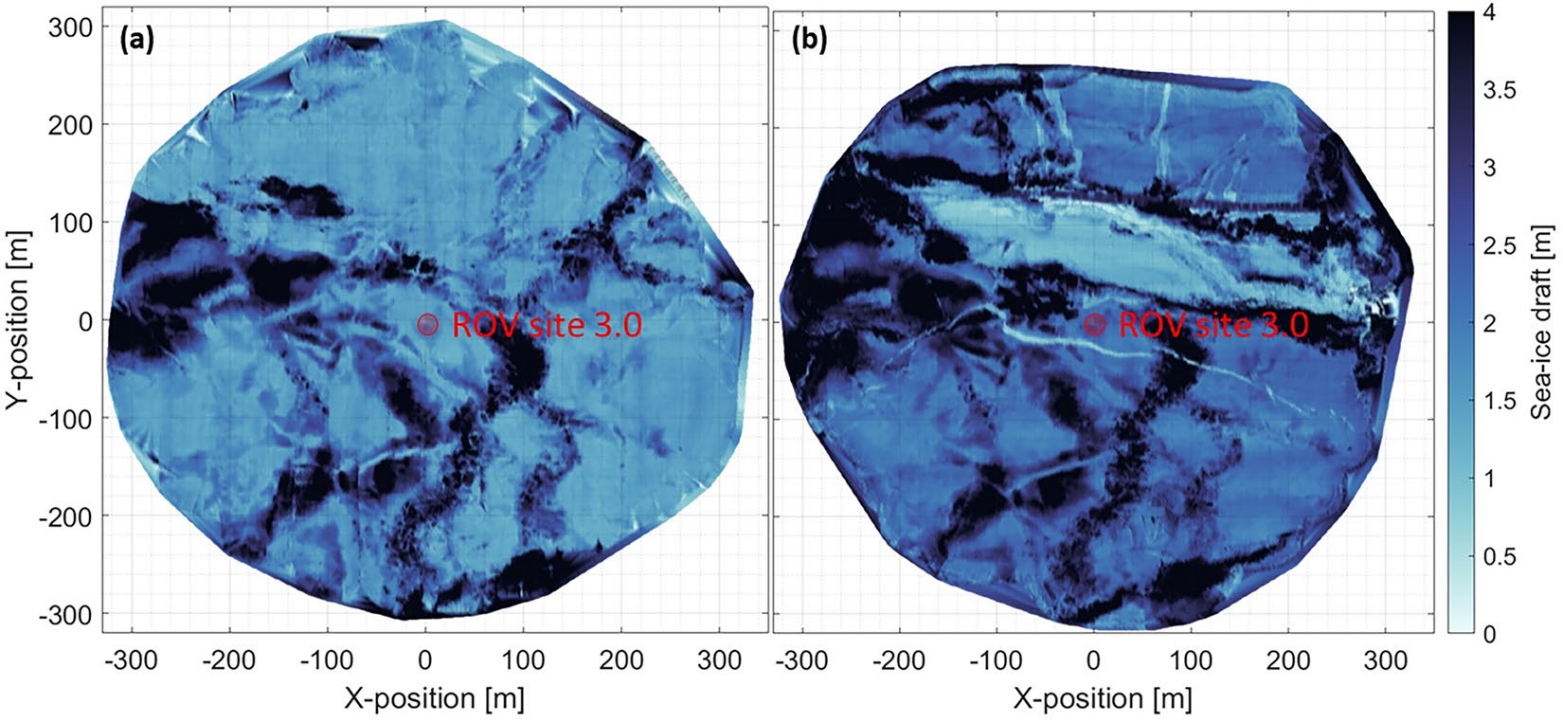
Map of the multibeam sea-ice draft on (**a**) 07/01/2020 and (**b**) 05/05/2020.

**Fig. 6:**
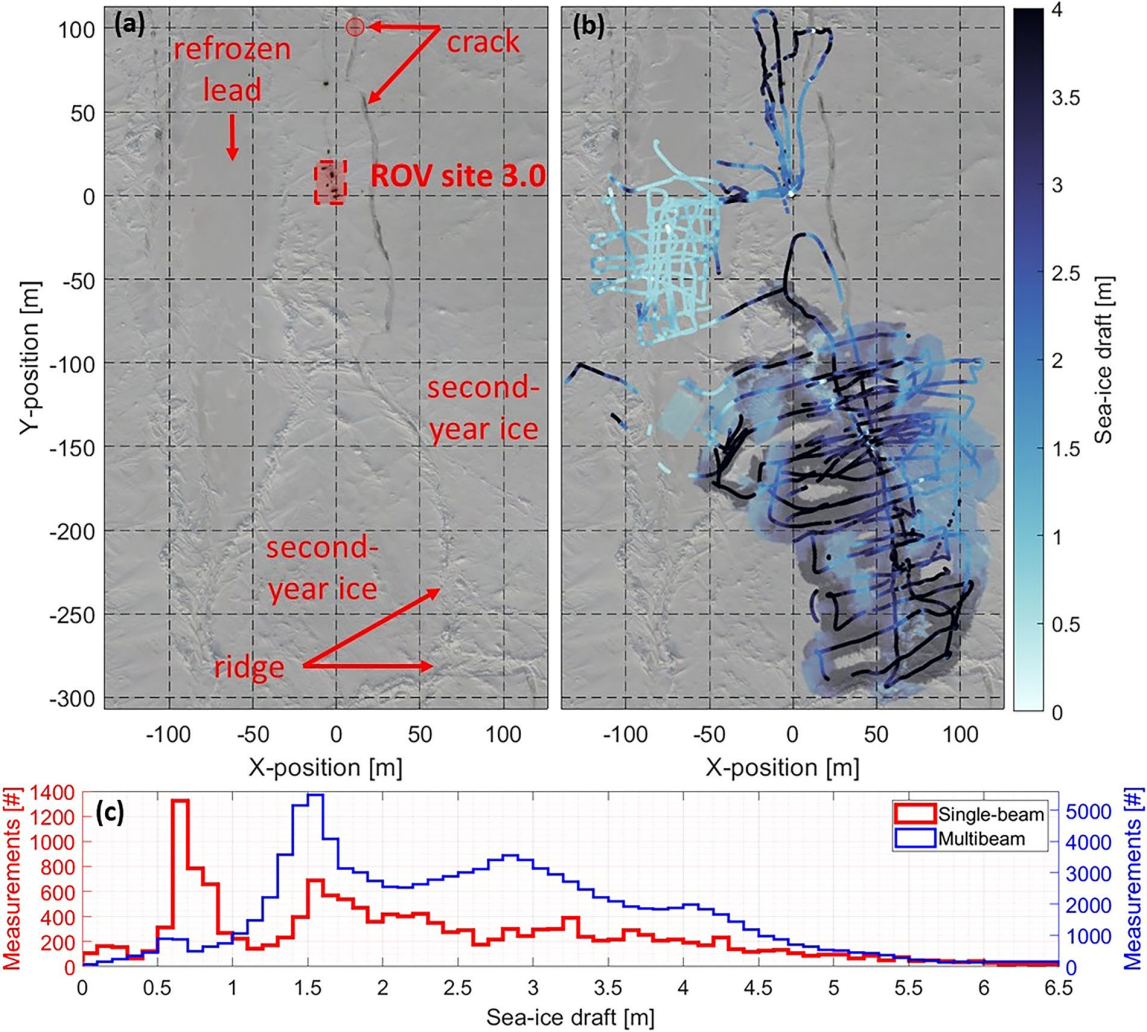
(**a**) RGB orthomosaic on 10/05/2020^82,98^. (**b**) Overlaid on the othomosaic is the sea-ice draft of second-year ice, a ridge, and a refreezing lead as measured on 09/05/2020 both by the altimeter (coloured dots) and the multibeam (coloured patch). (**c**) Corresponding sea-ice draft histograms.

Figure 5 shows maps of the sea-ice draft as derived from the multibeam sonar on 07/01/2020 (a) and on 05/05/2020 (b) for the maximum ROV extent of approximately 300 m. The temporal variability in terms of sea-ice deformation events that occurred between 07/01/2020 and 05/05/2020 is illustrated by a large refrozen lead and a long crack in Figs. 5b and 6a,b.

Examples of both the single-beam and the multibeam sea-ice draft as measured on 09/05/2020 are presented in Fig. 6. During that day, four ice types were covered: crack, refrozen lead, second-year ice, ridge. In the corresponding draft histograms (Fig. 6c), those ice types are characterized by modal draft values. The mode between 0.6 m and 0.7 m is associated with the refrozen lead. The modes between 1.5 m and 1.6 m and between 2.8 m and 2.9 m, the latter of which is more dominant in the multibeam draft, are associated with second-year ice. The tail of larger draft values is associated with the ridge. A smaller mode between 0.1 m and 0.2 m is associated with a recent opened and refrozen crack and is only represented in the single-beam data because the crack was not covered by the multibeam (Fig. 6a,b).

### Optical properties

**Fig. 7:**
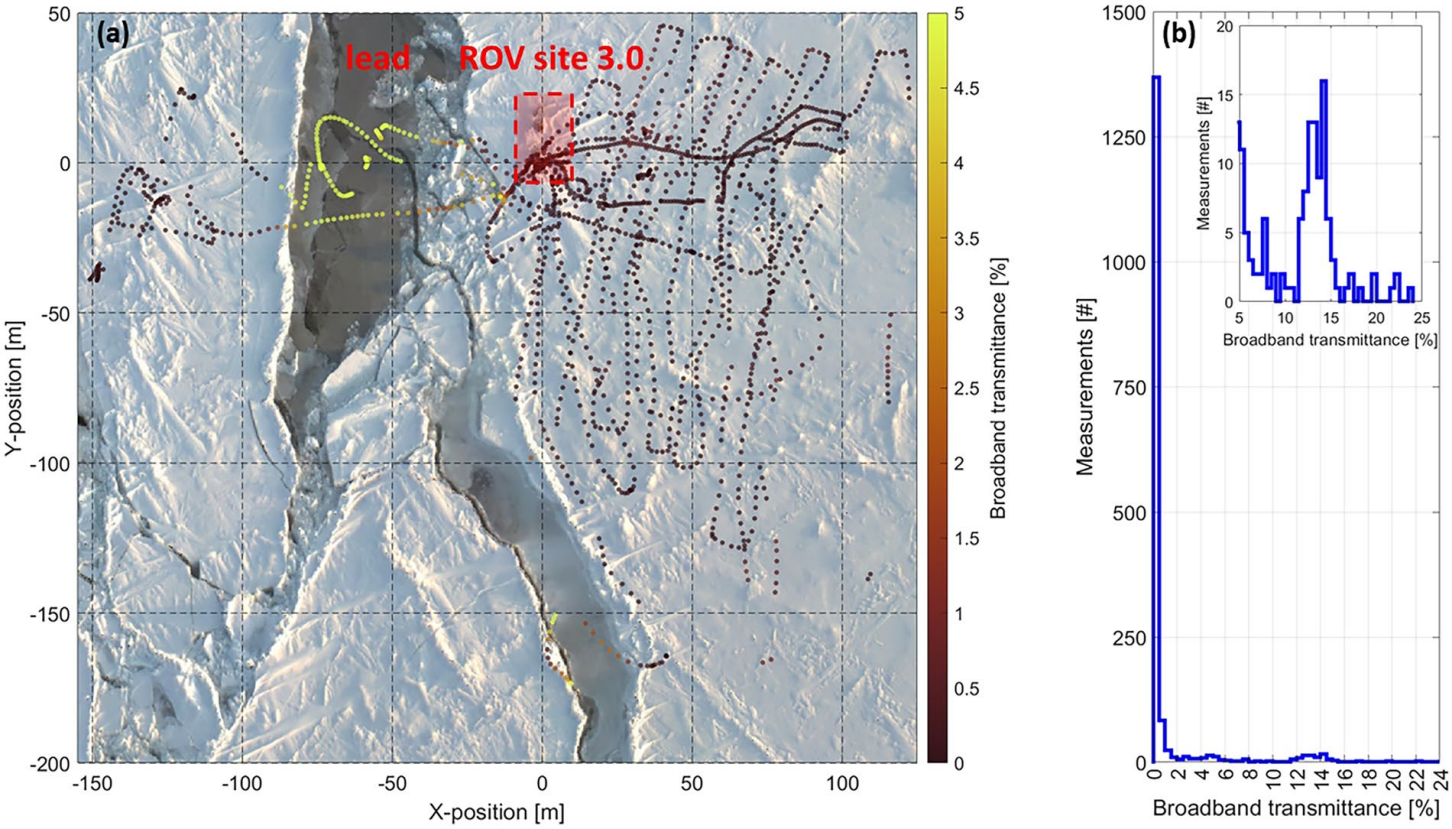
(**a**) Spatial variability of broadband light transmittance as obtained using the measurements from the irradiance radiometer attached to the remotely operated vehicle (ROV) and the surface irradiance radiometer on 22/03/2020. The background image is a RGB orthomosaic on 21/03/2020^82,98^. (**b**) Corresponding transmittance histogram.

The spatial variability of the broadband transmittance of a refreezing lead and of adjacent second-year ice on 22/03/2020 is presented in Fig. 7a. The transmittance of the refreezing lead is as high as 24% (Fig. 7b) at specific locations due to the very thin ice layer and partly still open water (Figs. 7a and 4c). The modal transmittance of the refreezing lead lies between 14% and 14.5% (Fig. 7b, inset). However, the overall most frequently measured transmittance value lies between 0% and 0.5% and, thus, is very low due to the thick second-year ice because the survey was concentrated underneath the second-year ice (Fig. 7a).

**Fig. 8:**
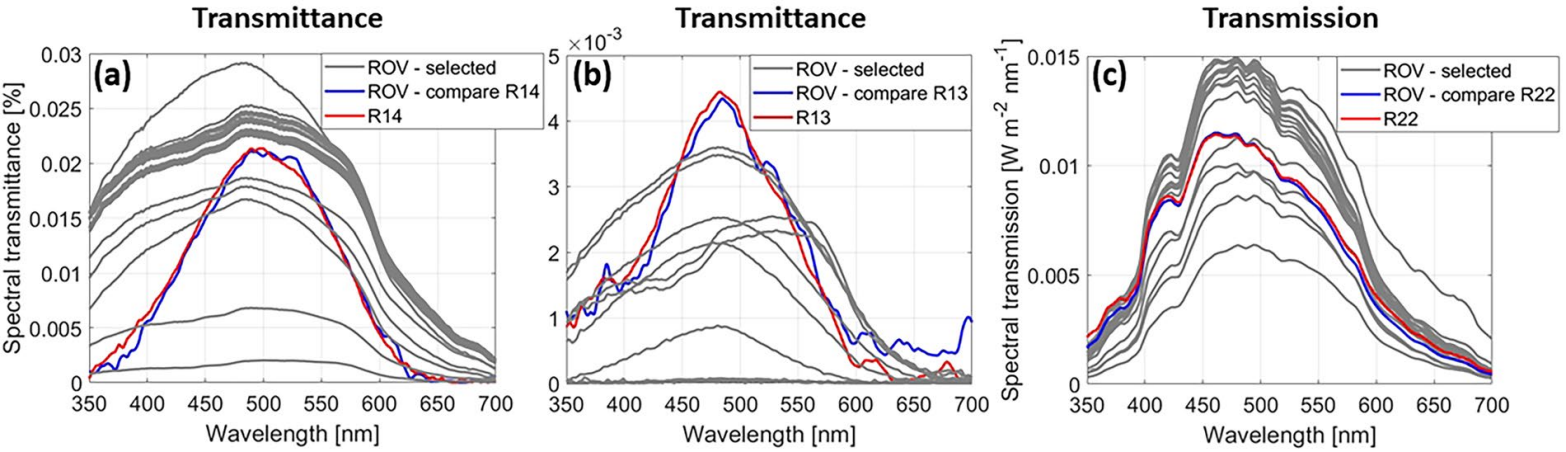
Comparison of transmittance and transmission spectra measured by the radiometers attached to the remotely operated vehicle (ROV) and to autonomous radiation stations^34,55^: (**a**) 02/05/2020, (**b**) 09/05/2020, and (**c**) 29/08/2020. Grey lines are selected ROV transmittance and transmission spectra for different ice types. Blue lines are mean ROV spectra measured at the radiation stations. Red lines are spectra from the radiation stations: 2020R14 (**a**) was deployed on second-year ice, 2020R13 (**c**) was deployed on a sea-ice ridge, 2020R22 (**d**) was deployed on level bare ice. The locations of the radiation stations are shown in Fig. 3a,c.

Examples of spectral transmittance and transmission as measured on 02/05/2020, 09/05/2020, and 29/08/2020 are presented in Fig. 8. The grey lines indicate spectra that have been randomly selected. Those spectra have different magnitudes and shapes as they have been measured underneath different ice types with varying properties such as thickness, snow depth, and in-ice inclusions. The blue lines denote temporal means (a few seconds) of spectra measured at specific locations where radiation stations were deployed (2020R13, 2020R14, and 2020R22 in Fig. 3a,c). The red lines represent spectra measured by the radiation stations. The comparison of the spectra measured by the ROV and by the radiation stations is discussed in the section Technical Validation. All spectra peak close to 500 nm where the sea ice is most translucent to solar radiation. The transmittance in (a) and (b) were measured underneath 3 m thick second-year ice and underneath a ridge more than 4 m thick, respectively, explaining their low magnitudes. The spectral transmission (Fig. 8c) was measured underneath 1.6 m level first-year ice. Thus, its shape is relatively smooth, besides the dip at around 440 nm which indicates the presence of biomass within the ice as at wavelengths between 430 nm and 440 nm the absorption coefficient of chlorophyll-a peaks^69,71,78^.

**Fig. 9:**
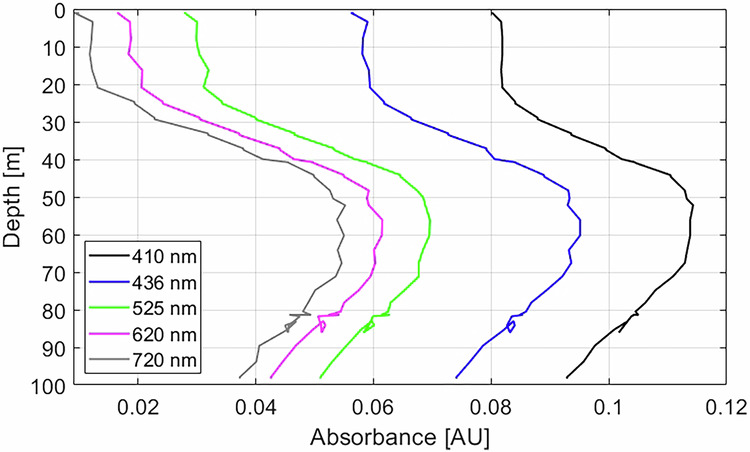
Depth profiles of absorbance at different wavelengths as obtained by the VIPER during the remotely operated vehicle (ROV) survey on 07/04/2020.

Depth profiles of the absorbance of light at different wavelengths collected on 07/04/2020 are presented in Fig. 9. The absorbance of light at wavelengths 410 nm and 525 nm was higher than the absorbance of light at 720 nm and 620 nm, respectively. This is an indicator for the presence of phytoplankton and zooplankton biomass in the water column that absorb visible light stronger at smaller wavelengths^79,80^. The maximum absorbance occurs at depths between 50 m and 70 m. This is in accordance with the echo sounder data collected onboard *Polarstern* that showed high abundance of scatters at those depth levels (personal communication: Serdar Sedan).

### Physical oceanographic properties

**Fig. 10:**
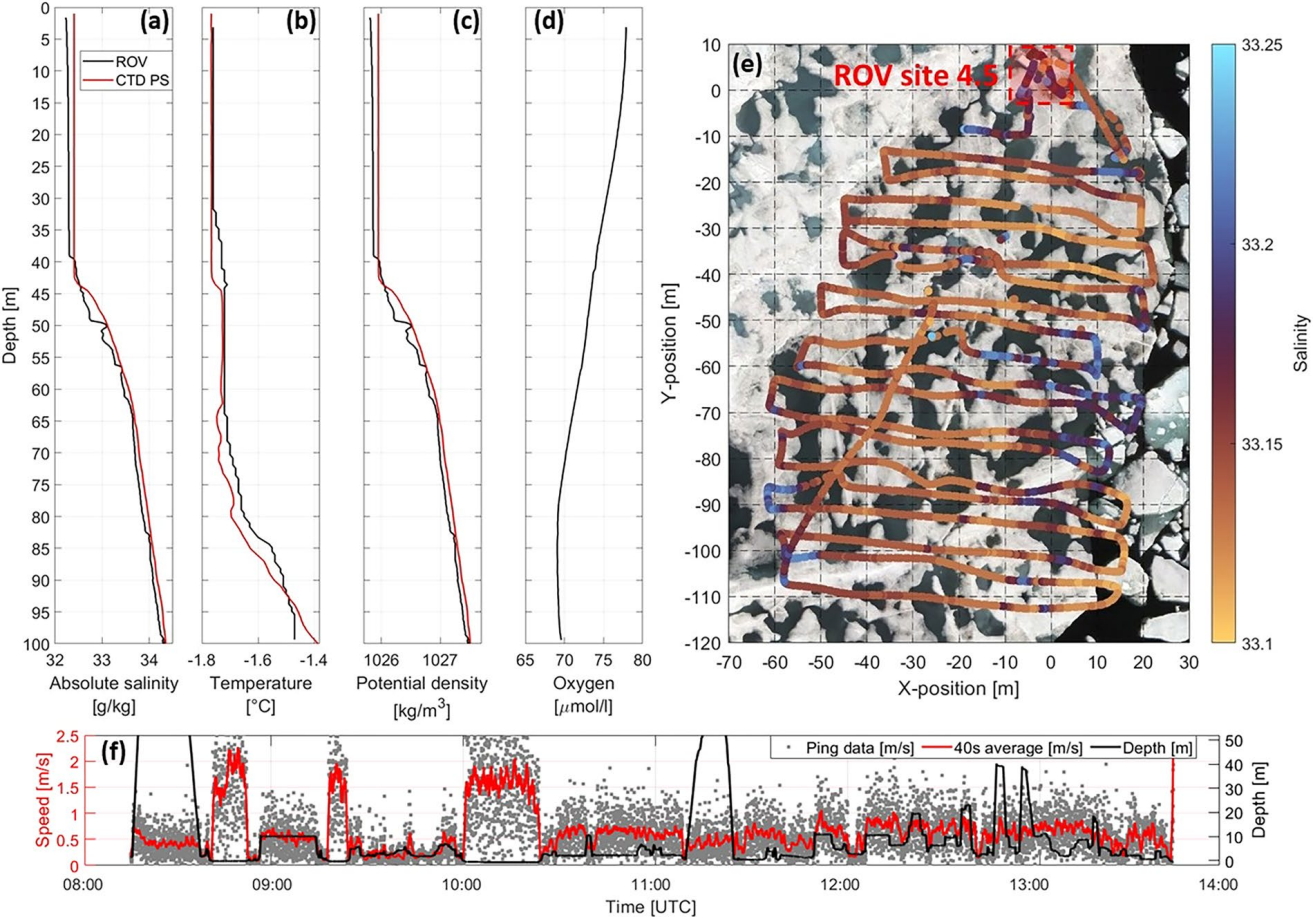
Depth profiles of (**a**) absolute salinity, (**b**) temperature, (**c**) potential density, and (**d**) oxygen as obtained from the GP-CTD during the remotely operated vehicle (ROV) survey on 22/02/2020 (black lines). The red lines show profiles obtained close to *Polarstern* (PS) using a large CTD rosette on 21/02/2020^38,93^ (Fig. 3a). (**e**) Horizontal variability of salinity at depths between 2.5 m and 3.5 m during the ROV survey on 14/07/2020. The background image is an RGB orthomosaic from 17/07/2020^82,98^. (**f**) Water current speed as obtained in the closest cell by the ADCP during the ROV survey on 07/04/2020.

Depth profiles of absolute salinity, temperature, oxygen, and potential density as obtained by the GP-CTD on 22/02/2020 are presented in Fig. 10a--d and the local variability in salinity in panel (e). The water in the upper 30 m of the water column is well mixed with relatively constant temperature, salinity, and density compared to water masses below. The salinity, and thus, the density, increases from 30 m to 40 m before a strong gradient in salinity at approximately 41 m prevails (pycnocline). While the temperature remains relatively constant until 80 m depth, the salinity increases continuously towards deeper depths. The oxygen concentration is higher at shallower depth close to the surface and decreases with depth towards 80 m. The spatial variability in salinity between 2.5 m and 3.5 m (Fig. 10e) is determined by the presence of fresh meltwater and salty seawater. Between 14/07/2020 and 16/07/2020 the occurrence of meltwater and the average freshwater equivalent thickness under the sea ice were high^81^. The freshwater resulted from ice and snow melt both by gradual percolation through the ice and by more rapid melt pond drainage events^81^.

The winter water temperature together with video footage and images were used to document the presence and distribution of platelet ice at the MOSAiC ice floe^57^. The analysis of this dataset shows that the subice platelet layer forms locally due to seed crystals in ocean surface supercooling^57^.

An example timeseries of speed through the water as obtained by the ADCP on 07/04/2020 is presented in Fig. 10f.

### Biological oceanographic properties

**Fig. 11:**
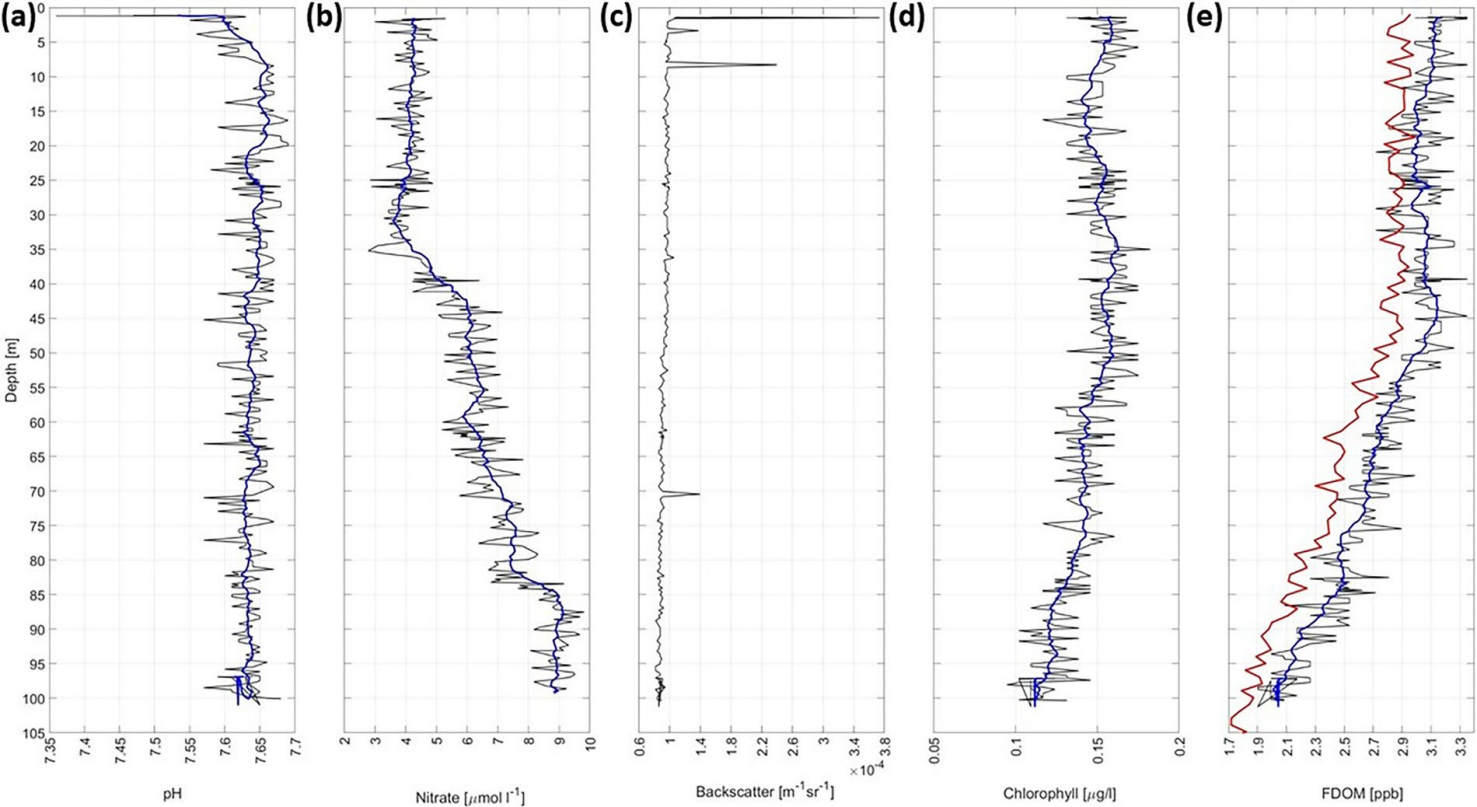
Depth profiles (black lines) of (**a**) pH values as obtained by the pH sensor, (**b**) nitrate concentration as obtained by the SUNA UV-spectrometer, (**c**) backscatter, (**d**) chlorophyll-a, and (**e**) FDOM (fluorescent dissolved organic matter) as obtained by the ECO-triplet fluorometer. Blue lines represent the smoothed data as obtained by applying a running mean with a window size of 10. The data was collected on 22/02/2020. The red line in (**e**) shows a FDOM profile obtained close to *Polarstern* using a large CTD rosette on 21/02/2020^38,93^ (Fig. 3a).

Figure 11 shows depth profiles of (a) pH values, (b) nitrate concentration, and (c-e) backscatter, chlorophyll-a, and FDOM as obtained on 22/02/2020. pH values of the seawater are constant throughout the examined water column at around 7.64, besides in the upper few meters close to the surface where the pH values are lower. The nitrate concentrations are with 4 *μ*mol l^−1^ between the surface and 30 m depth constant relative to concentrations below. Below 30 m, the concentrations increase continuously until 100 m depth where the concentration is 9 *μmol l*^−1^. Optical backscatter peaks close to the surface, at approximately 8 m, and at 70 m. However, those peaks are not represented in the chlorophyll-a or FDOM concentration. Both the chlorophyll-a and FDOM concentrations are relatively constant between the surface and 45 m before they continuously decrease with depth. Thus, the peaks in backscatter might be caused by larger animals. At those depths, the zooplankton sampling using the nets attached to the ROV were performed (Fig. 2b^39^), because a high abundance of zooplankton was expected at those depths. This sampling was performed to investigate the abundance, species composition, under-ice fauna, taxonomy, genetics as well as the foodweb structure of zooplankton and phytoplankton. A smaller net within the zooplankton net was used to collect gypsum in the water column^39,59^.

## Technical Validation

### Position

The quality of the ROV position was assessed by comparing the ROV positions at installations under the ice and their corresponding surface units as measured by the TLS with horizontal resolutions ≤ 0.02 m^47^ or as documented in helicopter-borne RGB orthomosaics with resolutions ranging between 0.03 m and 0.50 m^82^. The installations used were ROV markers and transponders (e.g., Fig. 2f), under-ice radiometers from radiation stations^34,55^, and other buoys deployed (e.g., Fig. 4f,g). The modal position uncertainty was 1.5 m based on mean uncertainties between ROV positions and respective installations during selected surveys.

**Fig. 12:**
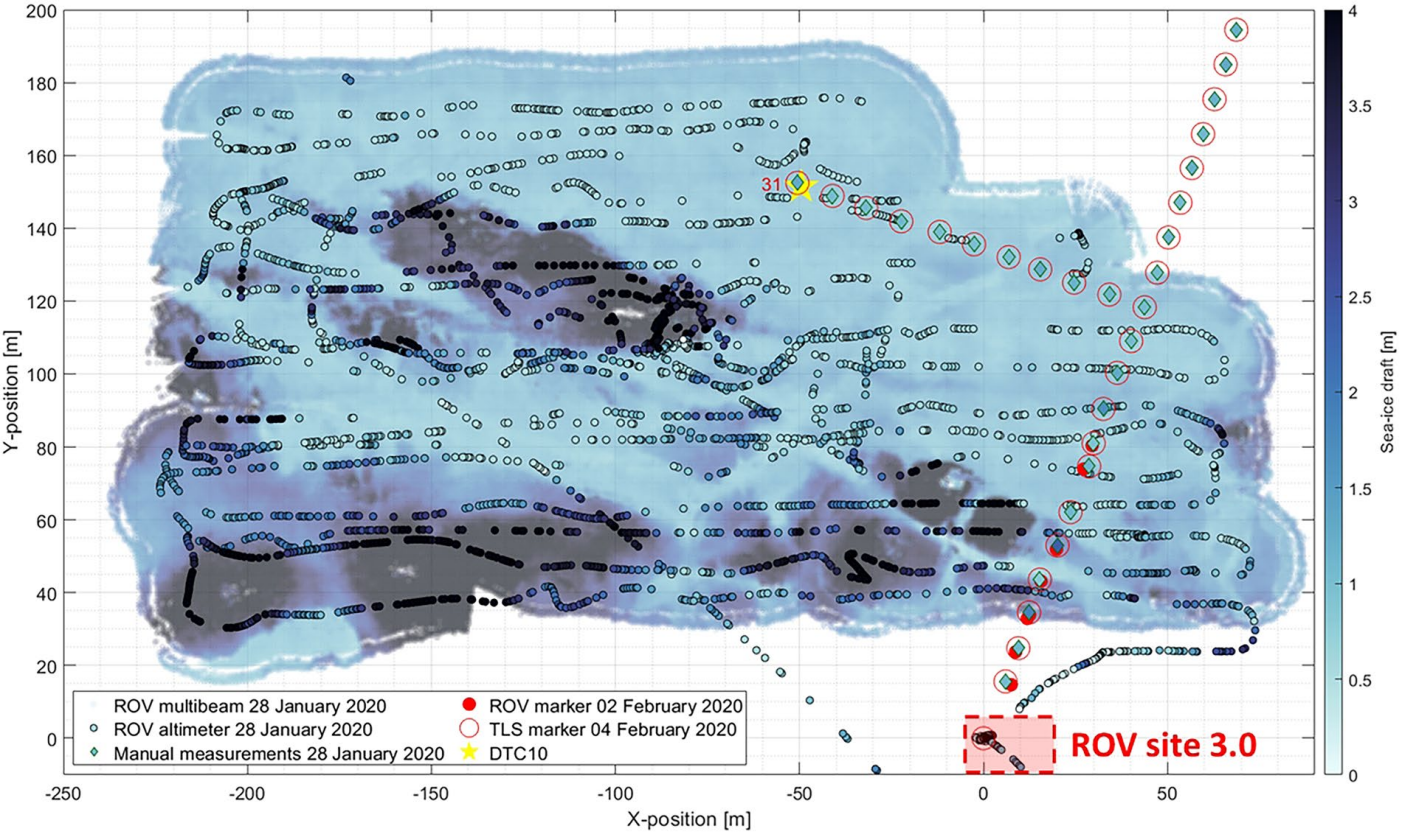
Map of multibeam and altimeter (black circled) sea-ice draft on 28/01/2020. Manual measurements from drillings, retrieved at the ROV marker locations (red circles), are indicated by the colored diamonds. The number 31 denotes the marker location at which the manual drilling was performed for comparison between sea-ice draft. The location of the digital thermistor chain (DTC10) is indicated by the yellow star.

A rough indication whether the ROV position was likely to be accurate to 1-2 m can be obtained by comparing the position under the ROV hole with upward-looking images and videos (e.g., Fig. 2e). For the comparison, favorably, ROV positions directly or very close to the markers were used. Example markers are shown in Figs. 2f,g and 12.

In general, the location of the transceiver of the ROV positioning system (placed in the back of the ROV) and the distance of installations to the HD camera lens (0.65 m), were taken into account in the position accuracy assessment. Due to strong drift, under-ice installations, especially ROV markers were sometimes shifted sidewards which translated into yet another position uncertainty. The TLS and orthomosaic coordinate systems were manually aligned to the ROV coordinate system by first referencing them to the ROV hole and second by rotation. Only ROV positions with a quality flag of “1” or “2” were used. Referencing to the ROV hole resulted in another uncertainty because the ROV hole was not a single and small point but an approximately 1.5 m x 1.5 m area and was covered by a tent during winter and spring. Figure 12 shows an example of how ROV marker positions scanned by the TLS were rotated to match ROV marker positions as documented by the ROV HD-zoom camera. To gain confidence in the choice of the rotation angle between ROV positions and corresponding positions in the orthomosaics, the broadband transmittance and the single-beam sea-ice draft as measured by the altimeter were used. For example, in Fig. 7a the spatial variability of the transmittance, e.g., the higher transmittance of thin ice of the refreezing lead makes sense, strengthen the choice of the rotation angle. In addition, the draft of a crack (Fig. 6a,b) is lower than the surrounding second-year ice, again giving confidence in the choice of the rotation angle. Yet another error source was based on the selection of installations in the orthomosaics that were snow covered or melted into the ice so that their positions could not be retrieved with high certainty. This is also connected to the resolution of individual orthomosaics. In general, the TLS measurements have a higher resolution resulting in better estimates of position uncertainties.

### Under-ice radiation

Under-ice irradiance measurements as obtained by the RAMSES radiometer attached to the ROV were validated by comparing those to measurements obtained from RAMSES radiometers attached to stationary radiation stations^34,55,83^–^85^. Selected mean spectra between ROV and radiation stations as compared when the ROV passed the stations are shown in Fig. 8. The spectral transmittances based on ROV and radiation stations 2020R14 and 2020R13 of second-year sea-ice and of a ridge in early May compare well despite being very low (Fig. 8a,b). The spectral transmissions as measured in late August compare also well (Fig. 8c). This comparison gives us confidence in the data quality of the ROV based radiation measurements.

The footprint radii from which the RAMSES radiometers detected radiation through the ice surface based on $R=D\times \tan (\beta /2)$^86^ varied with the distance D from the radiometers to the ice surface (including ice thickness) and the opening angles of the radiometers (Table 6). We assumed a 1:1 radius-ice thickness relationship, conservative scattering, and a diffuse light source. 50-60% of the photons reaching the radiometer originates from a 1.5-2.0 m footprint radius^87^. Errors to the radii are introduced by the pitch and roll of the ROV, which change the angle and surface area from which the radiometers detected radiation. Dedicated ROV optics surveys underneath level sea ice were usually carried out at 2 m water depth. Thus, for those surveys the footprint radii of the radiance and irradiance radiometers are 0.1 m and 115 m, respectively.

**Table 6:** Examples of footprint radii as a function of the opening angle of the radiometers (radiance or irradiance) and the distance to the sea-ice surface.

Distance to sea-ice surface [m]	Footprint radius [m] radiance radiometer (opening angle 7^∘^)	Footprint radius [m] irradiance radiometer (opening angle approximately 180^∘^)
0.1	0	5.7
1	0.1	57
1.5	0.1	86
2	0.1	115
5	0.3	286
10	0.6	573
50	3.1	2 865
100	6.1	5 729

### Sea-ice draft

The relative sea-ice draft for each individual survey derived from the ROV altimeter and multibeam sonar data is consistent as it reasonably reflects the structure of the sea-ice floes such as level ice, ridges, leads, and cracks (e.g., Figs. 5, 6, and 12). To validate the absolute sea-ice draft, we have compared the multibeam derived draft to 21 manual draft measurements from drillings and to derived draft from temperature measurements from digital thermistor chains (DTCs^88^), for both in January. The comparison reveals that the difference between the multibeam draft and the drafts derived from manual drillings and DTCs are larger than the accuracy of the ROV pressure/depth sensor of 0.10 m. However, the comparability of the multibeam draft values to point measurements in general comes with uncertainties due to the ROV position and the location of the point measurements which were co-located using TLS data^47^ and aerial images^82^.

For the comparison between multibeam draft and drafts obtained from point measurements (manual drillings and DTCs) all multibeam drafts within a 1.5 m radius around those points were averaged and compared to the draft at each respective point. The absolute mean and modal difference between multibeam drafts and 21 manual draft measurements for first-year ice on 28/01/2020 (Fig. 12) are 0.15 m and 0.16 m, respectively. The absolute mean and modal difference between multibeam drafts and the derived draft from DTC10 for first-year ice on 28/01/2020 (Fig. 12) are 0.18 m and 0.15 m, respectively. This comparison is the most reliable as multibeam, manual, and DTC drafts are available on the same date and for a level first-year ice patch.

The multibeam data has been used to investigate draft histograms to infer freezing rates for first-year ice, second-year ice, and a lead between end of December and beginning of May. The freezing rates are reasonable and comparable to the rates derived from heating rates of thermistor chains for the MOSAiC floe ranging from 0.004 m d^−1^ to 0.006 m d^−1^ depending on the initial ice thickness and snow depth^89^. The modal freezing rate based on the multibeam data for a level first-year ice patch for the period 31/12/2019 to 10/03/2020 (70 days) is 0.0071 m d^−1^ and for second-year ice for the period 07/01/2020 to 18/03/2020 (71 days) 0.0061 m d^−1^ and, thus, with approximately 0.01 m d^−1^ similar. The mean freezing rates are with 0.0051 m d^−1^ for first-year ice and 0.0058 m d^−1^ for second-year ice similar as well. This is surprising, as expected from the thermodynamic growth, thinner ice would grow faster than thicker ice. The ice of a refreezing lead between 31/03/2020 and 28/04/2020 (28 days) has grown much faster, at a rate of 0.015 m d^−1^ (mean and mode similar). Further, the multibeam data has been used to infer ridge keel melt rates^49^ and density estimates^90^ for summer sea ice.

While the direct comparison of absolute multibeam derived draft to other datasets yields an offset, both the relative drafts within each survey as well as the growth rates from December to May for different ice types appear reasonable. In the following, we discuss minor error sources that however cannot explain the presented differences but are discussed for completeness.

The error made in the retrieval of sea-ice draft from the multibeam data due to the usage of the constant sound speed in the processing scheme ranges between 0 m and 0.05 m and is thus within the accuracy of 0.10 m. We computed the sound speed from the ROV GP-CTD depth profile measurements using the equation stated in Table 4. The mean of the sound speed values between 0 m and 20 m depth was taken as a daily mean. From December towards mid of February, the daily mean of the sound speed was relatively equal. From mid of February until end of June, the sound speed increased continuously. In July and August, it varied but remained within the values that were measured in the months before. In September, it was again constant but much lower than throughout the rest of the year and than the constant value used for the multibeam data processing. The low September values can be explained by the relocation of *Polarstern* towards the North Pole where different water masses were present and sea-ice freeze-up had started.

Acoustic reflections at the ROV markers might have resulted in a few much higher draft values (Fig. 2f). Reflections (multiple) by platelet ice which was abundant in winter^57^ might be responsible for larger multibeam drafts while manual drafts might be lower because the ice thickness gauge has most likely penetrated the platelet ice layers. However, the error here is probably not larger than 0.10 m. Contrary to this argumentation is the effect of a fast-growing skeletal layer on the reflection. Acoustic beams from the multibeam might penetrate into the skeletal layer^91^ while the ice thickness gauge does not. This could have led to some underestimation of the multibeam draft when comparing to the manual draft. The high amount of brine that is rejected by refreezing leads and cracks can cause reflections inducing false distances between the multibeam and the under-ice surface. Such false reflections are hard to filter in the multibeam processing software. As for winter, a rapid evolving skeletal layer might also cause errors. The dependency of acoustic reflections of the sea-ice underside and the physical state of sea ice such as its temperature and porosity was verified in a laboratory setting^91^.

Another error source is based on how accurate the ROV operators performed the ROV depth calibration. It is expected that the error made can be at least 0.15 m. Lastly, atmospheric pressure variability adds additional error. Pressure changes were recorded by the Snow Buoy 2019S96^92^ from 29/10/2019 until 01/08/2020. During 21/04/2020, the air pressure change was especially large and has increased by 6.9 hPa from 1003.2 hPa at the start to 1010.1 hPa at the end of the ROV survey. This pressure change could account for a depth change of approximately 0.07 m. However, the error for most surveys is expected to be less as the mean atmospheric pressure variability was 0.88 hPa during the entire measurement period accounting for a mean depth change of less than 0.01 m.

The version 2.0 multibeam dataset published on PANGAEA (Table 3) was not corrected for the uncertainties and errors discussed here.

### Physical and biological oceanographic properties

A first validation of the physical and biological oceanographic properties obtained from the GP-CTD attached to the ROV was performed using data collected by a large CTD rosette operated from *Polarstern* (Fig. 3a^38,93^). The ice hole where the *Polarstern* CTD rosette was deployed into the ocean was located approximately 330 m away from the location where the ROV GP-CTD depth profile was conducted (Fig. 3a). This comparison data has been collected one day prior to the respective ROV survey. The different date and location on the ice floe add some uncertainties and differences to the comparison. However, the comparison shows that the ROV GP-CTD and the *Polarstern* CTD rosette data are similar.

The salinity, temperature, and density profiles (Fig. 10a–c) collected on 21/02/2020 by the *Polarstern* CTD rosette are similar to profiles collected by the ROV GP-CTD on 22/02/2020. The mean difference in absolute salinity between the ROV GP-CTD and the *Polarstern* CTD rosette is 0.10 g kg^−1^, ranging from 0 g kg^−1^ at 62 m to 0.32 g kg^−1^ at 55 m. The maximum difference is within two standard deviations of the ROV GP-CTD absolute salinity measurements, of all measurements around approximately  ±  2 m of the depth of the maximum difference. The mean difference in temperature between the ROV GP-CTD and the *Polarstern* CTD rosette is 0.01^∘^C, ranging from 0.002^°^C at 89 m to 0.080^°^C at 81 m. The maximum difference is within four standard deviations of the ROV GP-CTD temperature measurements, of all measurements around approximately  ±  2 m of the depth of the maximum difference. The mean difference in potential density between the ROV GP-CTD and the *Polarstern* CTD rosette is 0.08 kg m^−3^, ranging from 0 kg m^−3^ at 62 m to 0.26 kg m^−3^ at 55 m. The maximum difference is within two standard deviations of the ROV GP-CTD potential density measurements, of all measurements around approximately  ±  2 m of the depth of the maximum difference.

Fluorometric data on FDOM for comparison was obtained by a fluorometer (ECO FLRTD, WetLabs, Corvallis, OR, USA) attached to the *Polarstern* CTD rosette. The FDOM is generally lower but the shape of the profile is very similar, also the decrease towards deeper depth (Fig. 11e). The mean difference in FDOM between ROV GP-CTD and *Polarstern* CTD rosette is 0.2 ppb, ranging from 0 ppb at 71 m to 0.7 ppb at 82 m. The maximum difference is within three standard deviations of the ROV GP-CTD FDOM measurements, of all measurements around approximately  ±  2 m of the depth of the maximum difference. This is most likely due to calibration issues.

## Data Availability

The MATLAB scripts used to process the ROV data are available in Zenodo^75^ .
